# Influence of Cigarette Aerosol in Alpha-Synuclein Oligomerization and Cell Viability in SH-SY5Y: Implications for Parkinson’s Disease

**DOI:** 10.1021/acschemneuro.3c00771

**Published:** 2024-03-14

**Authors:** Yu-Xin Shen, Pe-Shuen Lee, Ming-Chu Teng, Jhih-Hong Huang, Chia C. Wang, Hsiu-Fang Fan

**Affiliations:** † Institute of Medical Science and Technology, National Sun Yat-sen University, Kaohsiung 804, Taiwan; ‡ Department of Chemistry, National Sun Yat-sen University, Kaohsiung 804, Taiwan; § Aerosol Science Research Center, National Sun Yat-sen University, Kaohsiung 804, Taiwan

**Keywords:** α-synuclein oligomerization, fluorescence correlation spectroscopy, particulate matter, cigarette aerosol, Parkinson’s disease, autophagy activity

## Abstract

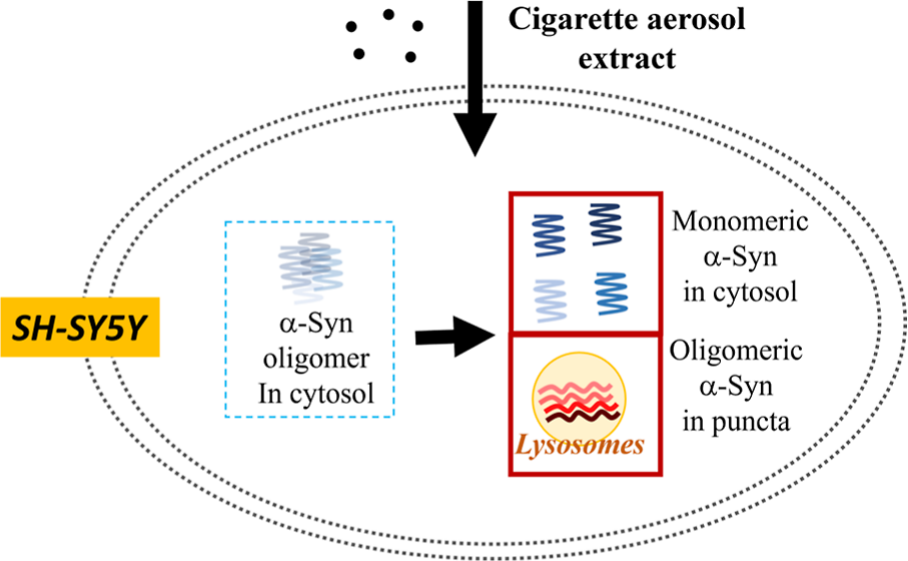

Although cigarette aerosol exposure is associated with various adverse health issues, its impact on Parkinson’s disease (PD) remains elusive. Here, we investigated the effect of cigarette aerosol extract (CAE) on SH-SY5Y cells for the first time, both with and without α-synuclein (α-Syn) overexpression. We found that α-Syn aggravates CAE-induced cell death, oxidative stress, and mitochondrial dysfunction. Fluorescence cross-correlation spectroscopy (FCCS) revealed a dual distribution of α-Syn within the cells, with homogeneous regions indicative of monomeric α-Syn and punctated regions, suggesting the formation of oligomers. Moreover, we observed colocalization of α-Syn oligomers with lysosomes along with a reduction in autophagy activity. These findings suggest that α-Syn overexpression exacerbates CAE-induced intracellular cytotoxicity, mitochondrial dysfunction, and autophagy dysregulation, leading to elevated cell mortality. Our findings provide new insights into the pathogenic mechanisms linking exposure to cigarette aerosols with neurodegenerative diseases.

## Introduction

PM2.5–10 exposure has been reported to increase pro-inflammatory and cancer biomarker levels in rat brains.^1^ Suspended particulate matter (SPM) contributes to neuroinflammation, cerebral vascular damage, impairment of the prefrontal cortex, and reduction in brain volume. Fine and ultrafine particulate matter affects the central nervous system through various pathways including (1) direct entry into the brain through the olfactory pathway; (2) indirect entry into the brain through the bloodstream after reaching the lungs; (3) induction of inflammation in the nasal and lung tissues by SPM, leading to the release of pro-inflammatory cytokines into the bloodstream and subsequent effects on the brain; and (4) activation of the hypothalamic–pituitary–adrenal axis, resulting in increased stress hormone (cortisol) levels in the bloodstream, thereby influencing the brain.^2−12^ A single-photon emission computed tomography study found higher deposition ratios of smaller particles in rat lungs.^13^ The deposition areas varied according to particle size:^14^ particles larger than 10 μm primarily deposited in the nasopharyngeal area, particles between 5 and 10 μm deposited in the bronchial area, and particles smaller than deposited 2.5 μm in the alveolar area.^15^ Particles smaller than 0.1 μm are more likely to enter the interstitial tissue than those smaller than 2.5 μm.^16,17^ Moreover, research also indicates that 15 nm aerosolized quantum dots, inhaled by mice, accumulate in the olfactory bulb and neuron axons, suggesting that small aerosols can impact the brain.^12^

Exposure to toxic aerosols or SPM, such as those from diesel engine exhaust or urban air pollution, can cause oxidative stress, DNA damage, neuroinflammation, and increased pro-inflammatory cytokines in rat brains, indicating brain inflammation.^18,19^ Moreover, a longitudinal study in Ontario from 2001 to 2013 revealed a 4% increase in Parkinson’s disease (PD) incidence per 3.8 μg/m^3^ increase in PM2.5,^20^ and short-term exposure to PM2.5 was reported to worsen symptoms in PD patients in Seoul.^21^ These studies suggest a strong association between aerosols and PD. Aerosols in cigarette smoke and air pollution share similar composition, including PAHs, heterocyclic compounds, nitrosamines, aromatic amines, aldehydes, phenolic compounds, hydrocarbons, nitroalkanes, metals, and other components,^22−24^ indicating a potential link between cigarette smoke aerosols and PD. Recently, the influence of nicotine, one of the substances present in tobacco smoke, on PD has been investigated, with some studies suggesting that it may delay PD progression in animal models,^25^ though conflicting findings also exist regarding its effects on PD’s progression.^26−30^ These observations indicate that the association between cigarette smoke and PD remains inconclusive.

While the link between cigarette smoke and Alzheimer’s disease (AD) has been extensively investigated,^31−33^ the relationship between cigarette smoke and PD remains unclear. In a previous study, we collected cigarette aerosols in a size-segregated manner, enabling a comprehensive assessment and comparison of the impacts exerted by cigarette aerosol extract (CAE) on biological systems.^34^ We used SH-SY5Y cells overexpressing α-synuclein (α-Syn) as a cellular PD model to investigate the effects of CAE exposure, monitoring changes in the oligomeric state of α-Syn using dual-fluorescence cross-correlation spectroscopy (FCCS). Employing live-cell imaging techniques, we aim to elucidate the potential pathological link between cigarette aerosols and PD progression.

## Results and Discussion

### α-Syn Overexpression Exacerbates CAE-Induced Cell Death, ROS Generation, and Mitochondrial Dysfunction in SH-SY5Y Cells

**Figure 1:**
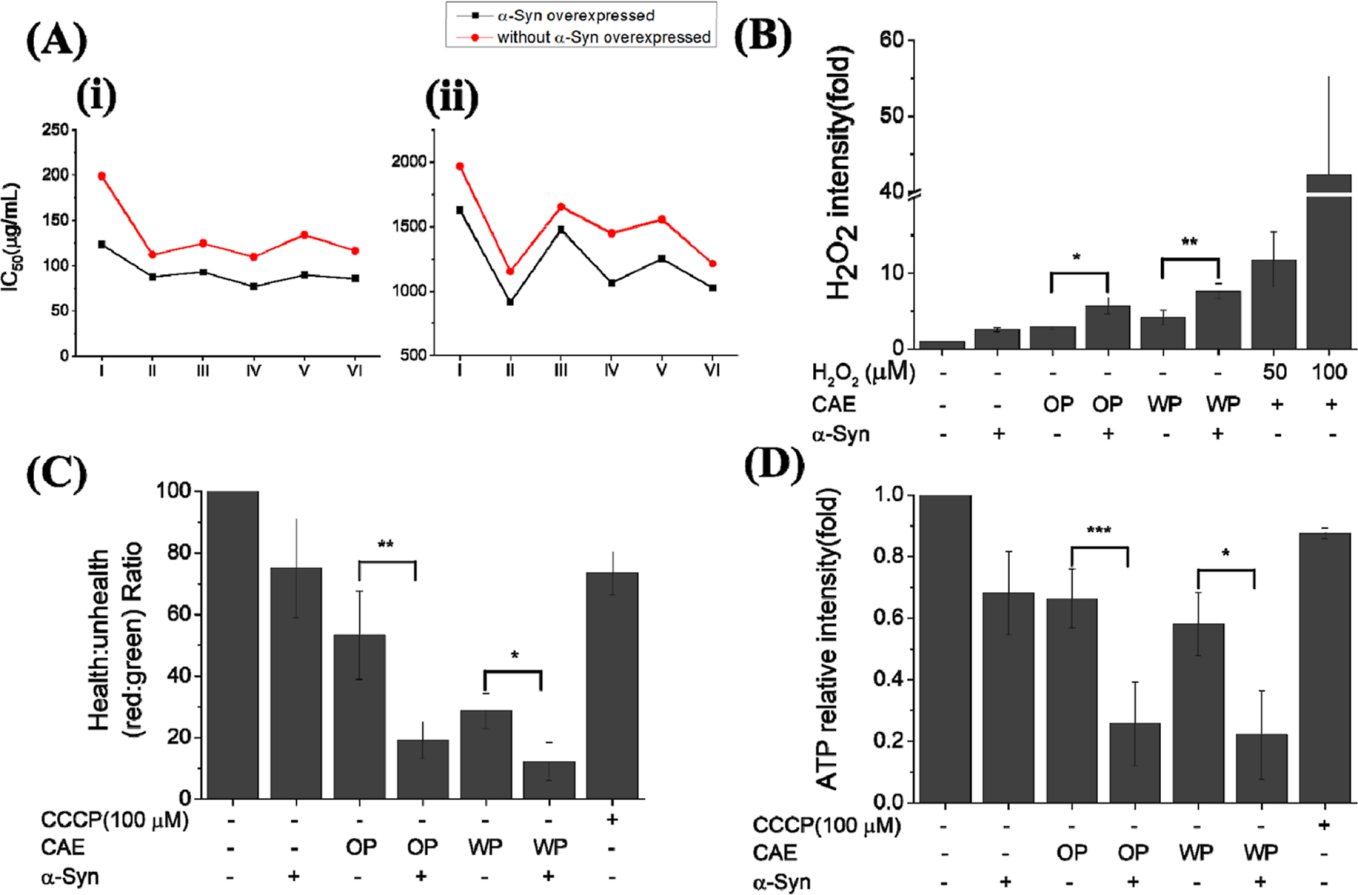
(A) Distribution of IC_50_ values from 3-repeated tests of CAE effects in the SH-SY5Y cell line with or without expressing WT α-Syn. The IC_50_ values of (i) OP extract and (ii) WP extracts. I∼VI indicate the size of cigarette aerosol obtained with MOUDI listed in Supporting Information Table 1. (B) Influence of cigarette aerosol extraction on intracellular H_2_O_2_ generation in the SH-SY5Y cell line with or without expressing WT α-Syn. The influences of cigarette aerosol extraction on (C) mitochondrial membrane potential polarization and (D) ATP generation in the SH-SY5Y cell line with or without expressing WT α-Syn. CCCP: Carbonyl cyanide 3-chlorophenylhydrazone. *, **, and *** represent significant differences (* = *p* < 0.05), (** = *p* < 0.01), and (*** = *p* < 0.001). The numbers indicate the number of investigated cells.

In our previous study, it has been reported that exposure of CAE decreases cell viability, increases intracellular reactive oxygen species (ROS) accumulation, and causes mitochondrial dysfunction.^34^ Moreover, the effects were most pronounced in SH-SY5Y among the three cell lines investigated.^34^ α-Syn is known for its role in the genetics and neuropathology of PD, with its misfolding and aggregation into Lewy bodies being pathological hallmarks of PD.^35−38^ Our previous research established a positive correlation between the concentration of intracellular α-Syn and enhanced cytotoxicity and reduced viability in SH-SY5Y cells.^39^ In this study, we used SH-SY5Y cells transfected with a plasmid to induce overexpression of α-Syn, a widely used cellular model for PD,^40−43^ to explore the potential impact of CAE on PD progression. We treated SH-SY5Y cells overexpressing α-Syn with various concentrations of CAE and assessed cell viability after 24 h. A significant decrease in cell viability (Figure S1) and a lower IC_50_ value (Figure 1A) were observed in cells overexpressing α-Syn and cocultured with CAE, indicating that α-Syn overexpression aggravates CAE-induced cell death. In contrast, cells overexpressing fluorescence protein and cocultured with CAE showed similar IC_50_ values to the control, suggesting that eGFP overexpression does not exacerbate CAE-induced cytotoxicity. Moreover, SH-SY5Y cells overexpressing eGFP-α-Syn and cocultured with CAE had comparable IC_50_ values to those overexpressing untagged α-Syn, further confirming the detrimental effect of α-Syn overexpression (Figure S2).

To investigate how α-Syn overexpression might exacerbate CAE-induced oxidative stress, we conducted experiments in SH-SY5Y cells overexpressing α-Syn for 24 h, followed by an 80 min treatment with organic particle (OP)–CAE or water-soluble particle (WP)–CAE to monitor intracellular H_2_O_2_ levels. Both α-Syn overexpression and CAE treatment independently increased intracellular H_2_O_2_ levels, but their combination led to an even more pronounced increase (Figure 1B) in α-Syn overexpression and CAE treatment. These findings indicate that overexpressing α-Syn further deteriorates CAE-induced oxidative stress.

Previous studies have shown mitochondrial dysfunction in the substantia nigra of PD patients, characterized by impaired mitochondrial complex I function^44,45^ and mitochondrial DNA deletions.^46^ Our previous experiments demonstrated decreased mitochondrial membrane potential and ATP levels in cells treated with CAE.^34^ We then assessed whether α-Syn expression exacerbated CAE-induced mitochondrial dysfunction. After 24 h of α-Syn overexpression followed by 150 min of CAE treatment, we observed a significantly pronounced decrease in the mitochondrial membrane potential using the JC-1 probe (Figure 1C). This result suggests that overexpressed α-Syn exacerbates CAE-induced mitochondrial dysfunction in SH-SY5Y cells.

Mitochondria are crucial for cellular energy production, generating adenosine triphosphate (ATP), and play a vital role in regulating the cell fate.^47,48^ Impairment in mitochondrial function can lead to reduced ATP synthesis, disrupted Ca^2+^ homeostasis, and excessive ROS production.^49^ We assessed cellular ATP levels in SH-SY5Y cells overexpressing α-Syn following CAE treatment and observed a considerably greater reduction in ATP levels than that under control conditions (Figure 1D). These findings indicate that α-Syn overexpression aggravated CAE-induced mitochondrial dysfunction, resulting in a substantial reduction in both mitochondrial membrane potential and cellular ATP levels.

### α-Syn Overexpression Exacerbates CAE-Induced Apoptosis and Pyroptosis but Attenuates CAE-Induced Autophagy Activity in SH-SY5Y Cells

Programmed cell death includes apoptosis, regulated necrosis, and autophagic cell death.^50,51^ Previous studies have demonstrated that cigarette smoke can induce cell death through various mechanisms such as pyroptosis,^52−55^ ferroptosis,^56−58^ autophagy,^59^ and apoptosis^60−63^ in various cell lines. In our prior study, we observed robust induction of caspase-1, caspase-3, and LC3-II activities upon exposure to CAE in multiple cell lines, indicating the activations of pyroptosis, apoptosis, and autophagy.^34^ This study aims to delve deeper into the synergistic effects of CAE treatment and α-Syn overexpression in SH-SY5Y cells.

**Figure 2:**
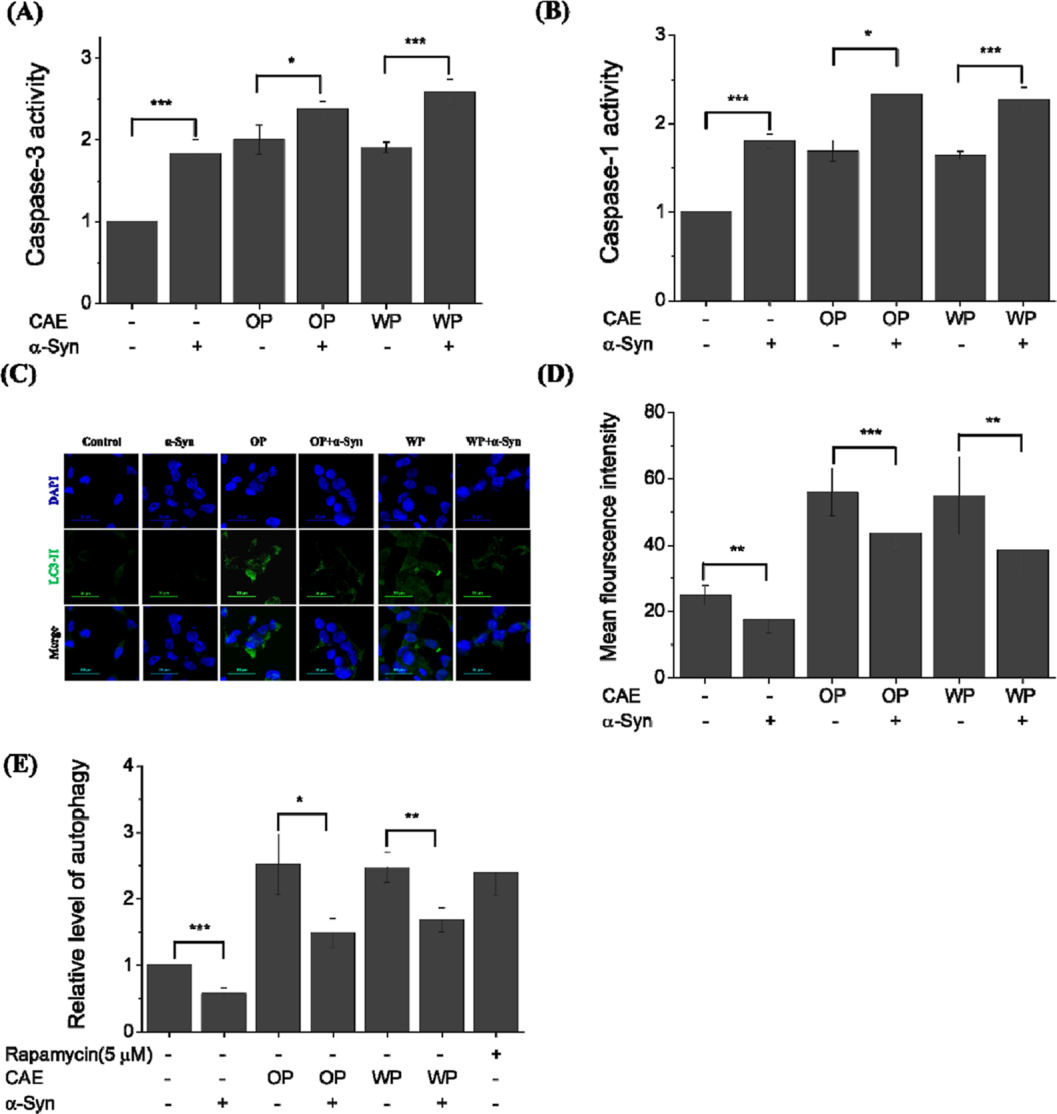
Impact of cigarette aerosol OP extraction on (A) caspase-1 and (B) caspase-3 activities and autophagy activities, as assessed through (C) LC3-II immunofluorescence images, (D) quantified LC3-II intensity derived from immunofluorescence images, and (E) autophagy activity assay analysis in SH-SY5Y cells, both with and without the expression of α-Syn. *, **, and *** represent significant differences (* = *p* < 0.05), (** = *p* < 0.01), and (*** = *p* < 0.001).

Here, an increase in Caspase-3 activity was observed in SH-SY5Y cells solely overexpressing α-Syn. Comparable increases in Caspase-3 activity were also observed in SH-SY5Y cells cocultured with either OP–CAE or WP–CAE. However, a more significant elevation in Caspase-3 activity occurred in cells overexpressing α-Syn and treated with OP–CAE or WP–CAE (Figure 2A, *p < 0.05 and **p < 0.01 against OP–CAE and WP–CAE treatment without α-Syn overexpression, respectively), suggesting an exacerbation of CAE-induced apoptosis due to α-Syn overexpression.

Similarly, an increase in Caspase-1 activity was observed in SH-SY5Y cells transfected with α-Syn alone and in those treated solely with CAE. This increase was more pronounced when α-Syn overexpressing cells were cocultured with OP–CAE or WP–CAE (Figure 2B, *p < 0.05 and **p < 0.01 against OP–CAE and WP–CAE treatment without α-Syn overexpression, respectively), indicating a potential intensification of CAE-triggered pyroptosis due to α-Syn overexpression.

Next, we investigate the impact of CAE treatment on autophagy levels in α-Syn overexpression in SH-SY5Y cells. Immunofluorescence staining to assess LC3-II expression, a marker of autophagy, revealed a notable reduction in autophagic response in both α-Syn-overexpressing and control groups (Figure 2C–E). This reduction contrasted with the elevated LC3-II fluorescence intensity in CAE-treated groups lacking α-Syn overexpression (Figure 2C–E). Furthermore, a marked decrease in autophagy levels was observed in α-Syn-overexpressing cells cotreated with CAE, compared to that in cells treated with CAE alone (Figure 2C–E, ***p < 0.001 and **p < 0.01 against OP–CAE and WP–CAE treatment without α-Syn overexpression, respectively). This indicates a dysregulation of autophagy caused by α-Syn overexpression in SH-SY5Y cells.

### CAE Treatment Alters the Oligomeric State of α-Syn and Induces the Formation of Punctate Structures in SH-SY5Y Cells

**Figure 3:**
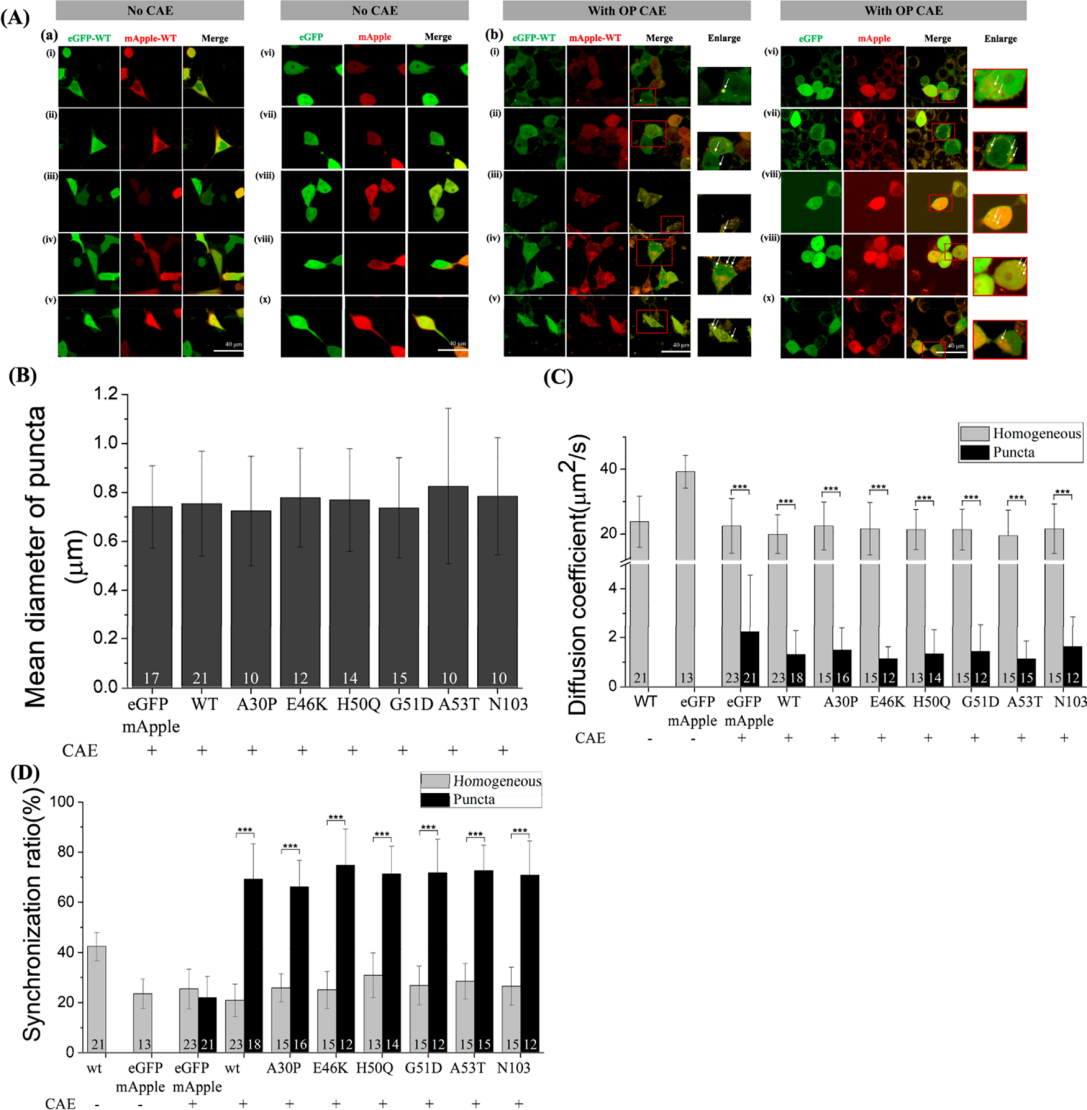
(A) Confocal images of SH-SY5Y cells expressing eGFP-α-Syn and mApple-α-Syn with or without treatment of OP CAE for 24 h. (B) Analysis of the mean diameter of puncta from SH-SY5Y cells expressing eGFP-α-Syn/mutant variants and mApple-α-Syn variants with or without treatment of OP CAE for 24 h. (C) Cross-correlation ratio of eGFP-α-Syn/mutant variants and mApple-α-Syn variants in SH-SY5Y cells with or without treatment of OP CAE for 24 h. (D) eGFP-α-Syn variants and mApple-α-Syn variants in SH-SY5Y cells with or without treatment of OP CAE for 24 h. Each FCS curve was fitted with the one-component 3D free diffusion model to obtain the corresponding diffusion coefficient (listed in Table 1). *, **, and *** represent significant differences (* = *p* < 0.05), (** = *p* < 0.01), and (*** = *p* < 0.001). The numbers indicate the number of investigated cells.

**Figure 4:**
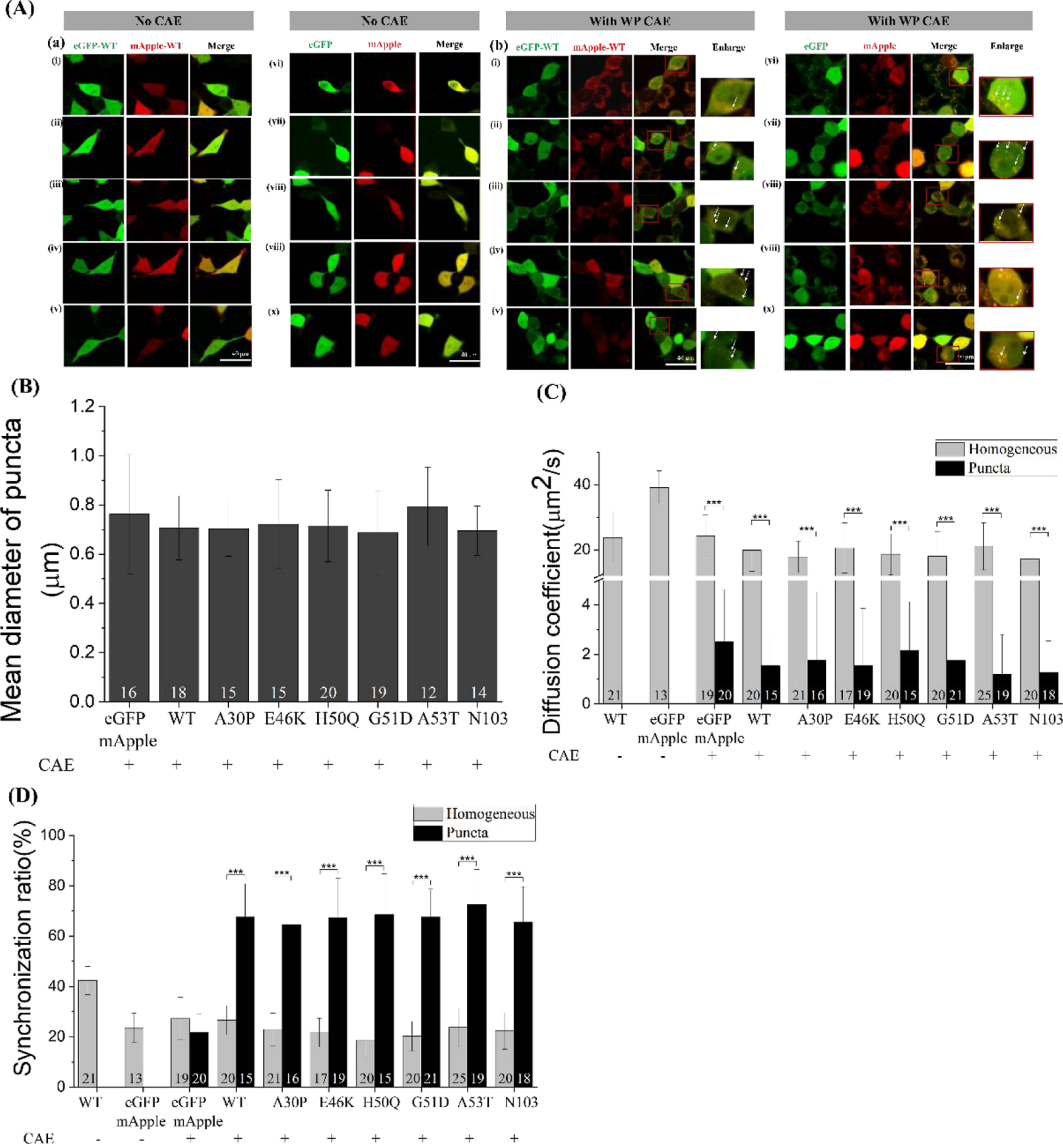
(A) Confocal images of SH-SY5Y cells expressing eGFP-α-Syn and mApple-α-Syn with or without treatment of WP CAE for 24 h. (B) Analysis of the mean diameter of puncta from SH-SY5Y cells expressing eGFP-α-Syn variants and mApple-α-Syn variants with or without treatment of WP CAE for 24 h. (C) Cross-correlation ratio of eGFP-α-Syn variants and mApple-α-Syn variants in SH-SY5Y cells with or without treatment of WP CAE for 24 h. (D) eGFP-α-Syn variants and mApple-α-Syn variants in SH-SY5Y cells with or without treatment of WP CAE for 24 h. Each FCS curve was fitted with the one-component 3D free diffusion model to obtain the corresponding diffusion coefficient (listed in Table 2). *, **, and *** represent significant differences (* = *p* < 0.05), (** = *p* < 0.01), and (*** = *p* < 0.001). The numbers indicate the number of investigated cells.

Previous studies have shown time-dependent oligomerization of α-Syn variants.^39^ In our study, we cocultured SH-SY5Y cells expressing eGFP-α-Syn variants and mApple-α-Syn variants with CAE samples (OP: 20 μg/mL and WP: 200 μg/mL) for 24 h to investigate the effect of CAE treatment. Without CAE treatment, we observed a homogeneous distribution of WT α-Syn variants without significant aggregation (Figures 3A_a and 4A_a), similar to previous findings.^39^ However, the coexpression of eGFP-α-Syn variants and mApple-α-Syn variants in the presence of CAE led to the formation of punctate structures (OP–CAE: Figures 3A_b and S3; WP–CAE: Figures 4A_b and S4). This punctate distribution was also observed in SH-SY5Y cells overexpressing untagged WT α-Syn when cocultured with CAE, suggesting that the punctate distribution is not a result of eGFP tagging (Figure S5). Interestingly, SH-SY5Y cells coexpressing eGFP and mApple also displayed similar punctate distributions in the presence of CAE (OP–CAE: Figure 3A_b; WP–CAE: Figure 4A_b). Subsequent imaging analysis revealed that the average diameter of these puncta was around 0.70 μm under all experimental conditions (OP–CAE: Figure 3B; WP–CAE: Figure 4B).

To further assess the impact of CAE treatment, we conducted repetitive fluorescence correlation spectroscopy (FCS) and FCCS measurements with a 60 s interval (Figures S3,S4). Without CAE treatment, diffusion coefficients for eGFP, eGFP-tagged WT α-Syn, and eGFP-mApple were 39.19 ± 5.10, 23.71 ± 7.97, and 36.10 ± 7.28 μm^2^/s, respectively, with concentrations <0.5 μM (OP–CAE: Figure 3C; WP–CAE: Figure 4C; and Tables 1 and 2), consistent with previously reported values.^39^ Following OP–CAE treatment, diffusion coefficients of eGFP significantly dropped to 22.46 ± 8.51 μm^2^/s in the homogeneous region and 2.22 ± 2.34 μm^2^/s in the puncta region (Figure 3 and Table 1). This decrease might be due to factors like changes in molecular weight and interactions with the cellular environment.^64−71^ To investigate further, a one-component anomalous diffusion model, commonly used to account for environmental crowdedness, intracellular interactions, and polydispersity behaviors of probe molecules,^64−71^ was used to analyze FCS curves. Anomalous diffusion modeling revealed increased hindrance, with an average anomalous factor of 0.74 ± 0.11 in the homogeneous region and 0.18 ± 0.14 in the puncta region compared to 0.91 ± 0.07 without CAE treatment (Figure 3 and Table 1). Lower cross-correlation ratios (25.50 ± 7.90 and 21.96 ± 8.53%) after OP–CAE treatment suggested that eGFP remained mostly monomeric, with the observed diffusion changes attributable to environmental factors. Similar results were obtained with the WP–CAE treatment (Figure 4 and Table 2).

**Table 1:** Diffusion Coefficients, Concentrations, and Anomalous Parameters of eGFP, mApple, eGFP-α-Syn Variants, and mApple-α-Syn Variants in SH-SY5Y Cells after 24 h of Transfection with OP CAE

	eGFP and mApple	eGFP WT & mApple WT	eGFP and mApple		eGFP WT & mAppleWT		eGFP A30P & mApple A30P		eGFP E46K & mApple E46K	
	without CAE (*N* = 3, *n* = 13)	without CAE (*N* = 4, *n* = 21)	type I (*N* = 6, *n* = 23)	type II (*N* = 5, *n* = 21)	type I (*N* = 7, *n* = 23)	type II (*N* = 5, *n* = 18)	type I (*N* = 3, *n* = 15)	type II (*N* = 3, *n* = 16)	type I (*N* = 3, *n* = 15)	type II (*N* = 3, *n* = 12)
diffusion coefficient (μm^2^/s) (free 3D diffusion fitting	39.19 ± 5.10	23.71 ± 7.97	22.46 ± 8.51	2.52 ± 2.09	19.85 ± 6.43	1.29 ± 0.98	22.43 ± 7.46	1.48 ± 0.92	21.58 ± 8.16	1.14 ± 0.50
eGFP concentration (nM)	100.45 ± 46.65	182.90 ± 97.63	167.77 ± 101.36	167.38 ± 132.10	178.48 ± 80.31	265.56 ± 144.71	308.78 ± 128.13	361.63 ± 162.76	265.39 ± 122.82	365.92 ± 192.81
mApple concentration (nM)	99.65 ± 64.13	151.84 ± 117.96	234.75 ± 133.26	158.48 ± 137.92	186.57 ± 162.95	166.69 ± 96.88	247.06 ± 105.84	316.46 ± 156.71	269.39 ± 135.92	260.76 ± 204.57
transport coefficient (μm^2^/s) (anomalous fitting)	41.63 ± 3.87 (α = 0.95 ± 0.06)	30.65 ± 7.66 (α = 0.81 ± 0.13)	36.84 ± 9.50 (α = 0.74 ± 0.11)	30.84 ± 9.05 (α = 0.18 ± 0.11)	35.17 ± 9.53 (α = 0.75 ± 0.10)	14.28 ± 10.65 (α = 0. ± 0.09)	35.07 ± 8.93 (α = 0.81 ± 0.08)	16.58 ± 8.98 (α = 0.26 ± 0.20)	31.06 ± 12.61 (α = 0.79 ± 0.16)	16.83 ± 7.44 (α = 0.23 ± 0.23)
FCCS ratio (%)	23.60 ± 5.77	42.37 ± 5.63	27.25 ± 8.41	25.50 ± 7.90	20.91 ± 6.47	69.25 ± 14.11	25.91 ± 5.61	66.18 ± 10.54	25.15 ± 7.31	74.76 ± 14.48

	eGFP H50Q & mApple H50Q		eGFP G51D & mApple G51D		eGFP A53T & mApple A53T		eGFP N103 & mApple N103		eGFP-mApple
	type I(*N* = 3,*n* = 13)	type II (*N* = 3, *n* = 14)	type I (*N* = 3, *n* = 15)	type II (*N* = 3, *n* = 12)	type I (*N* = 3, *n* = 15)	type II (*N* = 3, *n* = 15)	type I (*N* = 3, *n* = 15)	type II (*N* = 3, *n* = 12)	without CAE (*N* = 3, *n* = 13)
diffusion coefficient (μm^2^/s) (free 3D diffusion fitting	21.32 ± 7.03	1.33 ± 0.98	21.31 ± 6.32	1.42 ± 1.12	19.45 ± 7.97	1.13 ± 0.73	21.57 ± 7.75	1.62 ± 1.25	36.10 ± 7.28
eGFP concentration (nM)	348.04 ± 216.17	418.75 ± 186.35	266.48 ± 141.02	329.89 ± 170.73	248.17 ± 110.10	306.55 ± 167.22	213.38 ± 111.54	320.15 ± 217.30	177.54 ± 42.10
mApple concentration (nM)	374.81 ± 207.48	425.50 ± 234.98	379.15 ± 192.76	272.31 ± 210.87	334.06 ± 166.06	315.05 ± 273.26	231.16 ± 133.03	289.86 ± 165.71	137.80 ± 66.31
transport coefficient (μm^2^/s) (anomalous fitting)	30.38 ± 10.50 (α = 0.82 ± 0.06)	11.91 ± 0.60 (α = 0.30 ± 0.14)	31.07 ± 9.05 (α = 0.83 ± 0.15)	13.35 ± 8.44 (α = 0.38 ± 0.34)	33.69 ± 10.37 (α = 0.79 ± 0.16)	14.46 ± 11.84 (α = 0.20 ± 0.17)	38.68 ± 8.95 (α = 0.80 ± 0.05)	9.82 ± 4.67 (α = 0.46 ± 0.36)	39.71 ± 4.64 (α = 0.91 ± 0.04)
FCCS ratio (%)	30.93 ± 8.93	71.25 ± 11.05	26.87 ± 7.72	71.73 ± 13.52	28.54 ± 7.09	72.62 ± 10.21	26.56 ± 7.55	70.86 ± 13.62	55.54 ± 5.34

**Table 2:** Diffusion Coefficients, Concentrations, and Anomalous Parameters of eGFP, mApple, eGFP-α-Syn Variants, and mApple-α-Syn Variants in SH-SY5Y Cells after 24 h of Transfection with WP CAE

	eGFP&mApple	eGFP WT & mApple WT	eGFP and mApple		eGFP WT&mApple WT		eGFP A30P & mApple A30P		eGFP E46K & mApple E46K	
	without CAE (*N* = 3, *n* = 13)	without CAE (*N* = 4, *n* = 21)	type I (*N* = 3, *n* = 19)	type II (*N* = 3, *n* = 20)	type I (*N* = 3, *n* = 20)	type II (*N* = 3, *n* = 15)	type I (*N* = 3, *n* = 21)	type II (*N* = 3, *n* = 16)	type I (*N* = 3, *n* = 17)	type II (*N* = 3, *n* = 19)
diffusion coefficient (μm^2^/s) (free 3D diffusion fitting	39.19 ± 5.10	23.71 ± 7.97	24.20 ± 6.59	2.52 ± 2.09	19.85 ± 6.43	1.54 ± 1.31	17.81 ± 4.88	1.77 ± 2.76	20.59 ± 7.70	1.56 ± 2.30
eGFP concentration (nM)	100.45 ± 46.65	182.90 ± 97.63	192.39 ± 96.34	167.38 ± 132.10	152.62 ± 65.89	357.67 ± 238.02	125.51 ± 68.27	292.38 ± 158.25	128.29 ± 69.50	356.78 ± 153.22
mApple concentration (nM)	99.65 ± 64.13	151.84 ± 117.96	104.80 ± 64.76	158.48 ± 137.92	103.01 ± 46.15	163.82 ± 135.49	102.56 ± 56.34	181.2 ± 97.40	123.22 ± 100.58	138.57 ± 90.32
transport coefficient (μm^2^/s) (anomalous fitting)	41.63 ± 3.87 (α = 0.95 ± 0.06)	30.65 ± 7.66 (α = 0.81 ± 0.13)	33.98 ± 6.62 (α = 0.82 ± 0.29)	30.84 ± 9.05 (α = 0.18 ± 0.11)	34.12 ± 6.17 (α = 0.73 ± 0.29	9.15 ± 9.01 (α = 0.12 ± 0.05)	31.66 ± 7.30 (α = 0.79 ± 0.30)	11.70 ± 8.59 (α = 0.17 ± 0.09)	33.86 ± 8.61 (α = 0.71 ± 0.22)	15.15 ± 9.84 (α = 0.16 ± 0.08)
FCCS ratio (%)	23.60 ± 5.77	42.37 ± 5.63	27.25 ± 8.41	21.83 ± 7.26	26.63 ± 5.72	67.59 ± 13.25	22.89 ± 6.52	64.57 ± 15.81	21.63 ± 5.69	67.33 ± 15.65

	eGFP H50Q & mApple H50Q		eGFP G51D & mApple G51D		eGFP A53T & mApple A53T		eGFP N103 & mApple N103		eGFP-mApple
	type I (*N* = 3, *n* = 20)	type II (*N* = 3, *n* = 15)	type I (*N* = 3, *n* = 20)	type II (*N* = 3, *n* = 21)	type I (*N* = 3, *n* = 25)	type II (*N* = 3, *n* = 19)	type I (*N* = 3, *n* = 20)	type II (*N* = 3, *n* = 18)	without CAE (*N* = 3, *n* = 13)
diffusion coefficient (μm^2^/s) (free 3D diffusion fitting	18.62 ± 6.32	2.17 ± 1.96	18.03 ± 7.60	1.76 ± 2.26	21.10 ± 7.22	1.20 ± 1.60	17.20 ± 5.35	1.26 ± 1.29	36.10 ± 7.28
eGFP concentration (nM)	163.67 ± 158.45	243.59 ± 90.51	122.40 ± 57.52	225.20 ± 106.57	133.24 ± 83.17	261.74 ± 184.94	141.82 ± 81.63	252.90 ± 134.80	177.54 ± 42.10
mApple concentration (nM)	117.21 ± 87.74	136.54 ± 62.41	120.51 ± 73.50	164.23 ± 145.52	120.48 ± 44.09	117.99 ± 97.50	135.37 ± 96.88	109.95 ± 69.54	137.80 ± 66.31
transport coefficient (μm^2^/s) (anomalous fitting)	31.53 ± 6.41 (α = 0.74 ± 0.17)	11.66 ± 7.90 (α = 0.26 ± 0.08)	32.72 ± 9.89 (α = 0.79 ± 0.19)	11.15 ± 5.18 (α = 0.14 ± 0.05)	33.53 ± 6.99 (α = 0.72 ± 0.08)	11.37 ± 7.31 (α = 0.15 ± 0.11)	33.12 ± 8.17 (α = 0.72 ± 0.20)	13.58 ± 10.23 (α = 0.14 ± 0.03)	39.71 ± 4.64 (α = 0.91 ± 0.04)
FCCS ratio (%)	18.69 ± 6.35	68.57 ± 16.17	20.25 ± 5.80	67.57 ± 11.17	23.78 ± 7.55	72.55 ± 13.96	23.33 ± 7.26	65.57 ± 14.05	55.54 ± 5.34

Next, we examined the influence of the CAE on α-Syn variants. After OP–CAE treatment, eGFP-α-Syn variants in the homogeneous region showed no significant difference in diffusion coefficients compared to eGFP-WT α-Syn without CAE treatment (Figure 3 and Table 1). For eGFP-α-Syn variants in the homogeneous region after OP–CAE treatment, anomalous diffusion modeling revealed increased hindrance (an anomalous factor of 0.75–0.82) and FCCS experiments exhibited lower cross-correlation ratios of 20.91∼30.93%, indicating a monomeric state (Figure 3 and Table 1). In contrast, the puncta region showed a notable decrease in diffusion coefficients for the eGFP-α-Syn variants. Anomalous diffusion modeling suggested increased environmental hindrance with anomalous factors of 0.19–0.46 and local transport coefficients of 9.86–16.83 μm^2^/s^α^, and FCCS experiments revealed a higher synchronization ratio of 66.18∼74.76%, indicating the presence of oligomeric forms in this region after CAE treatment (Figure 3 and Table 1). Similar trends were observed with WP–CAE treatment, confirming that both treatments affected the oligomeric distribution of α-Syn in SH-SY5Y cells (Figure 4 and Table 2).

### CAE Treatment Leads to the Formation of α-Syn Puncta That Colocalize with Lysosomes in SH-SY5Y Cells

**Figure 5:**
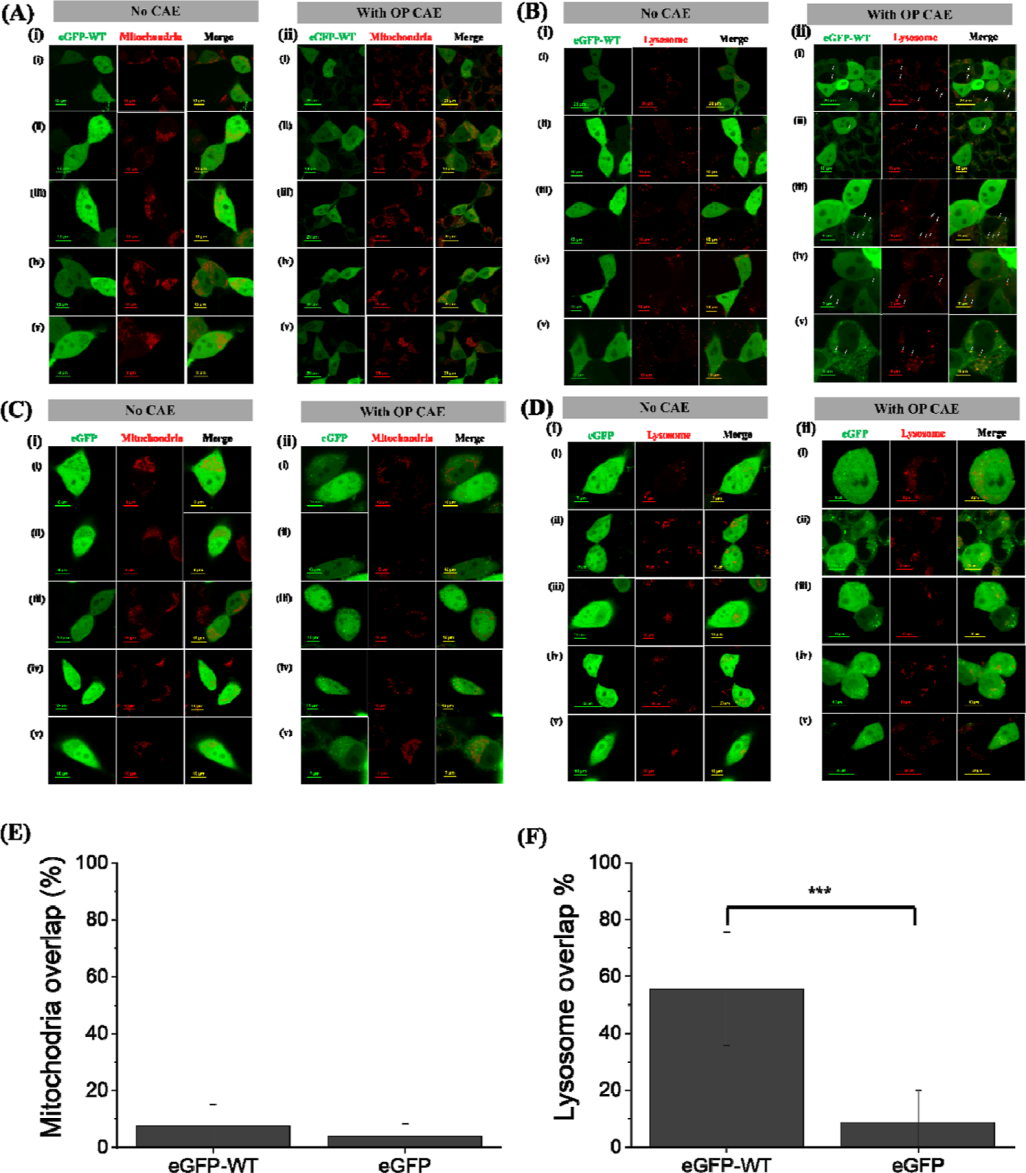
(A) Confocal images of SH-SY5Y cells expressing eGFP-WT α-Syn (green) and stained with MitoTracker (red) (i) without or (ii) with treatment of OP CAE for 24 h. (B) Confocal images of SH-SY5Y cells expressing eGFP-WT α-Syn (green) and stained with LysoTracker (red) (i) without or (ii) with treatment of cigarette aerosol OP extraction for 24 h. (C) Confocal images of SH-SY5Y cells expressing eGFP (green) and stained with MitoTracker (red) (i) without or (ii) with treatment of OP CAE for 24 h. (D) Confocal images of SH-SY5Y cells expressing eGFP (green) and stained with LysoTracker (red) (i) without or (ii) with treatment of cigarette aerosol OP extraction for 24 h. Quantified analysis of colocalization of eGFP-WT α-Syn and eGFP with (E) mitochondria or (F) lysosomes. Arrows indicate the overlapping region. *, **, and *** represent significant differences (* = *p* < 0.05), (** = *p* < 0.01), and (*** = *p* < 0.001).

Following CAE treatment, significant punctal structures were observed. This led us to investigate potential intracellular structures that might overlap with these intracellular oligomeric α-Syn puncta. Initially, we used MitoTracker Deep Red FM, a fluorescent dye for live mitochondria, to assess whether the intracellular oligomeric α-Syn puncta colocalize with mitochondria. SH-SY5Y cells expressing eGFP α-Syn were cocultured with OP–CAE for 24 h, and mitochondrial localization was stained with MitoTracker Deep Red FM. The resulting images showed α-Syn distribution in green and mitochondrial localization in red (Figure 5A). The merged image, appearing yellow, indicated that the α-Syn puncta induced by OP–CAE did not associate with mitochondria (Figure 5A).

Previous study has suggested a significant overlap between α-Syn and LAMP-2 protein, implicating lysosomal degradation of α-Syn.^72^ Subsequently, we examined whether CAE-induced oligomeric α-Syn puncta colocalize with lysosomes in SH-SY5Y cells. Using LysoTracker Red DND-99, we observed a significant overlap between the oligomeric α-Syn puncta and lysosomes, suggesting an association (Figure 5B). Control experiments with SH-SY5Y cells expressing eGFP and treated with OP–CAE for 24 h showed no significant overlap of eGFP puncta with either mitochondria or lysosomes (Figure 5C,D). This is consistent with the observed differences in the diffusion behavior and oligomeric state within puncta regions between eGFP and eGFP-α-Syn variants after CAE treatment. Quantification of the overlap between intracellular oligomeric α-Syn puncta and mitochondria or lysosomes was performed by using ImageJ software (Figure 5E,F). The analysis revealed an overlap percentage of 8.78 ± 7.70% for mitochondria and 54.38 ± 21.62% for lysosomes. This significant difference suggests that CAE-induced intracellular oligomeric α-Syn puncta are predominantly associated with lysosomes, implying that CAE-induced α-Syn puncta might affect the lysosome-dependent regulation mechanism.

## Discussion

It has been documented that α-Syn can interact with ATP synthase, disrupting ATP production, as well as interact with the mitochondrially encoded cytochrome *c* oxidase II (MT-CO2) protein, leading to increased production of ROS and the release of cytochrome *c*, ultimately promoting cell apoptosis.^73−76^ Moreover, α-Syn may bind with the mitochondrial adenine nucleotide translocator, facilitating the opening of membrane transport proteins and causing a decrease in the mitochondrial membrane potential, leading to mitochondrial dysfunction and promoting cell apoptosis.^73,74,77^ In this study, we investigated the synergetic effects of CAE treatment and α-Syn overexpression in SH-SY5Y cells. We found that α-Syn overexpression exacerbates CAE-induced intracellular cytotoxicity, accumulation of ROS, and mitochondrial dysfunction, resulting in increased cell death. The extent of neuronal cell death in PD patients has been positively correlated with the proportion of activated caspase-3-positive neurons.^78^ Furthermore, both fibrillar and monomeric forms of α-Syn have been reported to induce complete NLRP3 inflammasome activation, leading to caspase-1 activation and IL-1β production.^79^ In our study, substantial escalations in both Caspase-1 and Caspase-3 activities were observed in SH-SY5Y cells overexpressing α-Syn and cocultured with CAE. This suggests that α-Syn overexpression might potentiate CAE-induced cell death through the activation of pyroptosis and apoptosis.

In the FCCS experiments, we noted reduced diffusion coefficients for eGFP and eGFP-α-Syn variants in SH-SY5Y cells cocultured with CAE, compared to previous findings.^39^ Moreover, anomalous factors in homogeneously distributed regions ranged from ∼0.74 to 0.83 for eGFP and eGFP-α-Syn variants after CAE treatment (Figures 2 and 3 and Tables 1 and 2), indicating a significant increase in environmental hindrance due to CAE treatment. Previous research has shown that PM_10_ exposure in A549 cells increases filamentous actin (F-actin) presence by 50%.^80^ Nicotine has been reported to stimulate the release of mitogens, such as the platelet-derived growth factor, affecting the cell cytoskeleton.^81^ Furthermore, volatile organic components in cigarette aerosols, like acrolein and acetate, have been observed to alter the cytoskeleton in human gingival cells.^82^ Based on these studies, it is reasonable to infer that coculturing SH-SY5Y cells with CAE induces changes in the cell cytoskeleton, potentially leading to the entrapment of eGFP and eGFP-α-Syn variants within specific organelles and a decrease in diffusion coefficients, consistent with our observations here.

Epidemiological investigations have shown an inverse relationship between cigarette smoking and the progression of PD.^83−86^ However, conflicting reports suggest that cigarette smoking has either no association^87,88^ or is strongly linked with PD-related psychosis^89^ and cognitive impairment.^90,91^ Therefore, the relationship between cigarette smoking and PD remains controversial. In our previous study, we found that eGFP-α-Syn variants formed oligomers without CAE treatment, in line with prior research.^92,93^ However, when cocultured with CAE, we observed increased FCCS ratios (66.18–74.76%) in puncta regions, resembling those in the positive control group (55.55 ± 5.34%, Table 1, eGFP-mApple without CAE). Conversely, in homogeneously distributed regions, FCCS ratios decreased to about 20.9–30.9%, akin to the negative control group (23.60 ± 5.77%, Table 1, eGFP and mApple without CAE). Lower FCCS ratios in homogeneous regions suggest a shift toward monomeric structures of α-Syn, while higher FCCS ratios in puncta regions indicate α-Syn oligomerization. Prior studies have reported that nicotine exhibits antifibrillogenic and fibril-destabilizing activities toward the α-Syn aggregation process, which has implications for the prevention and treatment of Lewy body diseases.^94−96^ It is intriguing to explore whether CAE treatment similarly slows down the oligomerization rate of α-Syn in SH-SY5Y cells, resulting in the monomeric form observed here after 24 h of transfection and cotreatment with CAE. Extending the coculture and transfection period to 48 h showed no significant change in FCCS ratios and diffusion coefficients (Figures S6 and S7 and Table 3), suggesting that CAE facilitates the transition of α-Syn into monomeric structures rather than inhibiting oligomerization. Previous research indicates that WT α-Syn exists in a stable tetrameric form, while missense mutations tend to form monomeric structures that trigger pathological aggregation.^97^ Moreover, the disruption or impairment of α-Syn multimerization leads to an excess of monomers at vesicle membranes, resulting in round cytoplasmic inclusions and indicating pathological consequences.^98−101^ Our results suggest that CAE disrupts the α-Syn oligomeric equilibrium, favoring monomers with hindrance in homogeneous regions and causing oligomer formation in punctate distributions in SH-SY5Y cells. This finding highlights the potential link between CAE and α-Syn pathology.

**Table 3:** Diffusion Coefficients, Concentrations, and Anomalous Parameters of eGFP, mApple, eGFP-WT, and mApple-WT in SH-SY5Y Cells after 48 h of Transfection with OP CAE and WP CAE

	eGFP WT & mApple WT		eGFP and mApple				eGFP WT&mApple WT			
	without CAE		type I		type II		type I		type II	
	24 h (*N* = 4, *n* = 21)	48 h (*N* = 3, *n* = 13)	24 h (*N* = 6, *n* = 23)	48 h (*N* = 3, *n* = 13)	24 h (*N* = 5, *n* = 21)	48 h (*N* = 3, *n* = 14)	24 h (*N* = 7, *n* = 23)	48 h (*N* = 3, *n* = 18)	24 h (*N* = 5, *n* = 18)	48 h (*N* = 3, *n* = 12)
diffusion coefficient(μm^2^/s) (free 3D diffusion fitting	23.71 ± 7.97	16.00 ± 4.18	22.46 ± 8.51	15.90 ± 7.35	2.22 ± 2.34	2.29 ± 1.56	19.86 ± 6.06	16.26 ± 8.51	1.29 ± 0.98	1.40 ± 1.03
eGFP concentration (nM)	182.90 ± 97.63	383.52 ± 155.80	167.77 ± 101.36	371.87 ± 212.67	198.09 ± 139.96	280.04 ± 144.29	178.48 ± 80.31	290.02 ± 139.15	265.56 ± 144.71	963.68 ± 423.20
mApple concentration (nM)	151.84 ± 117.96	2241.2658.50 ±	234.75 ± 133.26	327.72 ± 216.97	222.45 ± 120.57	178.81 ± 167.15	186.57 ± 162.95	232.35 ± 177.29	166.69 ± 96.88	759.80 ± 423.20
transport coefficient (μm^2^/s) (anomalous fitting)	30.65 ± 7.66 (α = 0.81 ± 0.13)	19.44 ± 5.49 (α = 0.76 ± 0.13)	36.84 ± 9.50 (α = 0.74 ± 0.11)	34.38 ± 6.89 (α = 0.72 ± 0.14)	33.26 ± 9.92 (α = 0.18 ± 0.04)	31.82 ± 14.33 (α = 0.17 ± 0.08)	35.17 ± 9.53 (α = 0.75 ± 0.10)	33.10 ± 8.95 (α = 0.74 ± 0.19)	14.28 ± 10.65 (α = 0.19 ± 0.09)	10.57 ± 9.96 (α = 0.15 ± 0.07)
FCCS ratio (%)	42.37 ± 5.63	60.52 ± 6.44	25.50 ± 7.90	17.75 ± 6.84	21.96 ± 8.53	17.09 ± 7.38	20.91 ± 6.47	19.90 ± 7.22	69.25 ± 14.11	67.52 ± 12.28

	eGFP and mApple				eGFP WT&mApple WT			
	type I		type II		type I		type II	
	24 h (*N* = 3, *n* = 19)	48 h (*N* = 3, *n* = 14)	24 h (*N* = 3, *n* = 20)	48 h (*N* = 3, *n* = 15)	24 h (*N* = 3, *n* = 20)	48 h (*N* = 3, *n* = 19)	24 h (*N* = 3, *n* = 15)	48 h (*N* = 3, *n* = 19)
diffusion coefficient(μm^2^/s) (free 3D diffusion fitting	24.20 ± 6.59	18.17 ± 7.05	2.52 ± 2.09	3.28 ± 2.94	19.85 ± 6.43	16.29 ± 6.36	1.54 ± 1.31	1.93 ± 1.75
eGFP concentration (nM)	192.39 ± 96.34	254.87 ± 129.88	167.38 ± 132.10	310.54 ± 212.43	152.62 ± 65.89	262.23 ± 178.60	357.67 ± 238.02	694.99 ± 395.08
mApple concentration (nM)	104.80 ± 64.76	104.05 ± 70.03	158.48 ± 137.92	268.48 ± 306.14	103.01 ± 46.15	251.11 ± 218.63	163.82 ± 135.49	529.37 ± 372.38
transport coefficient (μm^2^/s) (anomalous fitting)	33.98 ± 6.62 (α = 0.82 ± 0.29)	33.03 ± 9.04 (α = 0.79 ± 0.11)	30.84 ± 9.05 (α = 0.18 ± 0.11)	37.27 ± 13.85 (α = 0.26 ± 0.10)	34.12 ± 6.17 (α = 0.73 ± 0.29)	36.00 ± 5.34 (α = 0.75 ± 0.13)	9.15 ± 9.01 (α = 0.12 ± 0.05)	14.65 ± 10.2 (α = 0.16 ± 0.06)
FCCS ratio (%)	27.25 ± 8.41	23.38 ± 7.91	21.83 ± 7.26	21.14 ± 7.90	26.63 ± 5.72	23.46 ± 5.23	67.59 ± 13.25	73.24 ± 11.93

Significant colocalization between CAE-induced α-Syn oligomeric puncta and lysosomes was observed in SH-SY5Y cells overexpressing α-Syn and cocultured with CAE. This suggests that CAE-induced α-Syn puncta might affect lysosome-dependent regulatory mechanisms. The lysosome plays an important role in mediating the autophagy process to maintain cellular homeostasis.^102^ Abnormal lysosome activity or autophagy dysregulation can lead to the aggregation of misfolded α-Syn and subsequent neuronal cell death, implying that the autophagy process may play an important role in neurodegenerative diseases, such as PD.^72,102−106^ Although CAE treatment induces autophagy activity, a decreasing trend in autophagy levels was observed in SH-SY5Y cells overexpressing α-Syn and cocultured with CAE, compared to that in cells treated with CAE alone (Figure 2C–E). Interestingly, the decreased autophagy activity was not observed in SH-SY5Y cells overexpressing eGFP and cocultured with CAE (Figure S8), further confirming α-Syn influence. This suggests a dysregulation of autophagy levels when α-Syn-overexpressing cells are exposed to CAE, highlighting the interplay between α-Syn pathology and autophagy dysregulation.

**Figure 6:**
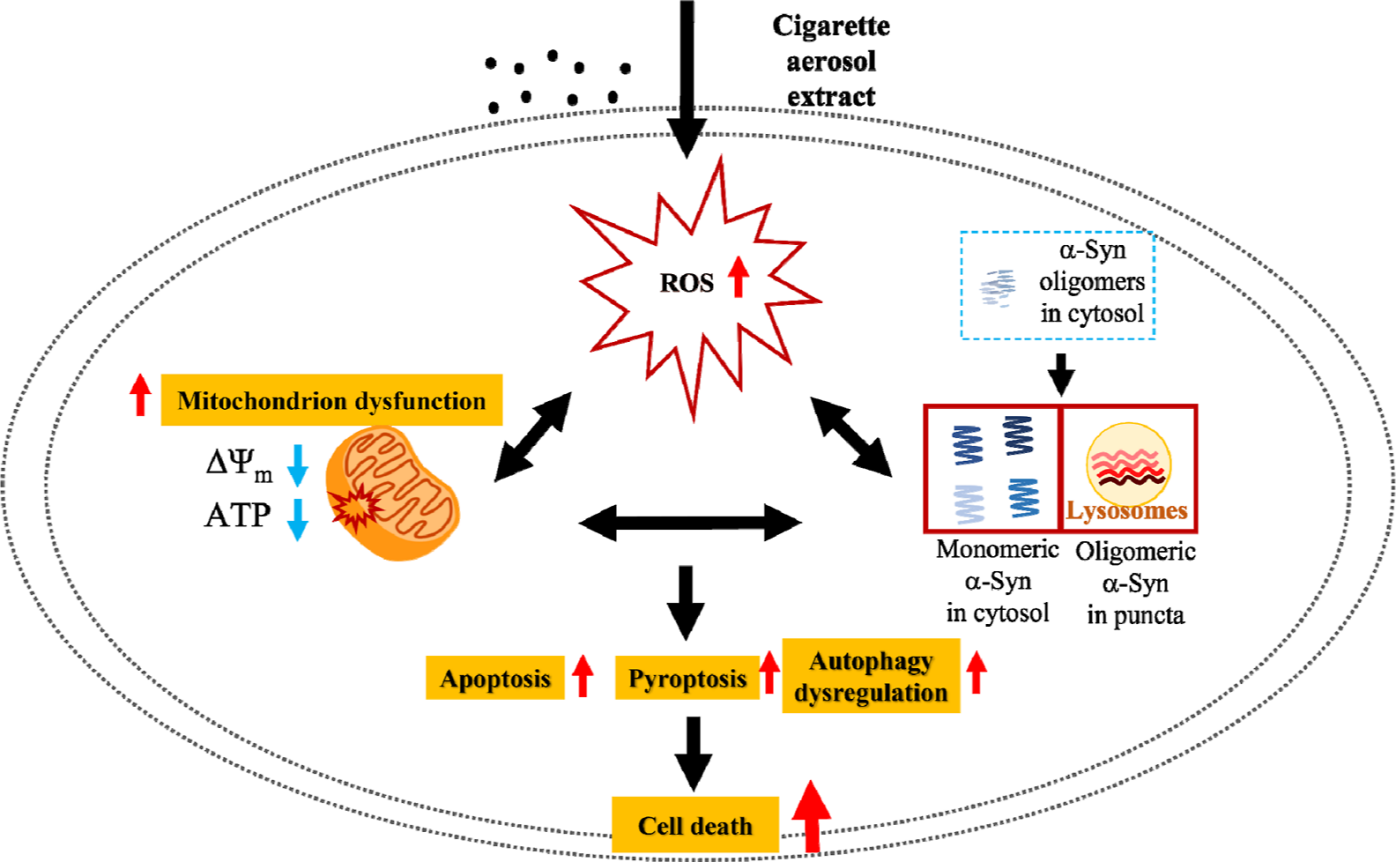
Summary mechanism of CAE-induced cell death in SH-SY5Y cells expressing α-Syn (description given in the Discussion section).

In conclusion, our study provides compelling evidence that α-Syn overexpression exacerbates CAE-induced intracellular cytotoxicity, leading to the accumulation of ROS and mitochondrial dysfunction. These cellular abnormalities ultimately result in significant cell death through apoptosis and pyroptosis. Moreover, we observe dysregulation of autophagy levels in α-Syn-overexpressing cells exposed to CAE, highlighting the intricate complex interplay between α-Syn pathology and autophagy dysregulation. CAE treatment disrupts the dynamic equilibrium in the oligomeric state of α-Syn variants, shifting native oligomers to monomers suffering significant environmental hindrance in homogeneously distributed regions and causing oligomeric α-Syn variants in punctate distributions colocalized with lysosomes in SH-SY5Y cells. These findings shed light on the molecular mechanisms underlying α-Syn-associated neurodegenerative disorders and the impact of CAE on α-Syn pathology (Figure 6).

## Materials and Methods

### DNA Constructs for the Live-Cell Studies

In our study, we used a peGFP-C1 vector to express eGFP in SH-SY5Y cells. The eGFP-α-Syn variants were constructed by ligating α-Syn into the peGFP-C1 vector using XhoI and Hind III restriction sites. Site-directed mutagenesis was employed to introduce mutations, resulting in the creation of Egfp-A30P, Egfp-E46K, Egfp-H50Q, Egfp-G51D, and Egfp-A53T. The eGFP-N103 construct was similarly generated by ligating N103 into peGFP-C1 using XhoI and Hind III. For the expression of mApple in SH-SY5Y cells, the pmApple-C1 construct was used. Following the same procedures as with eGFP, we constructed mApple-α-Syn, mApple-A30P, mApple-E46K, mApple-H50Q, mApple-G51D, mApple-A53T, mApple-N103, and eGFP-mApple.^39^

### Cigarette Smoke Aerosol Collection and Preparation of Its Extracts

A locally popular cigarette brand (Long Life Gentle 6, Taiwan), containing 5 mg of tar and 0.4 mg of nicotine per cigarette, was burned in a homemade smoking chamber. The resulting cigarette aerosol was collected using a 10-stage Micro-Orifice Uniform Deposit Impactor (Model 120 MOUDITM II Impactor, USA) at a pump flow rate of 30 m^3^ min^–1^ (GAST, 1023–101Q-SG608X). The collected aerosol samples were categorized based on particle size into different stages. The filters containing aerosol of various diameters were extracted with ddH_2_O and ethyl acetate to obtain water-soluble components (WP) and organic components (OP), respectively.^34^ The extracted samples were then dried and stored at −80 °C for further experiments on cytotoxicity and toxicology.^34^

### Cell Culture and Transfection

SH-SY5Y cells were cultured in high-glucose Dulbecco’s modified Eagle’s medium (DMEM, Gibco, 11965–084) supplemented with 1% penicillin/streptomycin (Gibco, 15140–122) and 10% (v/v) fetal bovine serum (FBS, Corning, 35–010-CV). The cells were maintained in 10 cm culture dishes (α-plus, 16203–1SS) at 37 °C in a saturated humidity atmosphere containing 95% air and 5% CO_2_. For assays measuring cell viability, ROS generation, mitochondrial function verification, caspase 1/3 activity, and autophagy activity, SH-SY5Y cells were transfected with a specific amount WT α-Syn for 24 h using TurboFect (Thermo Fisher, R0531), following the vendor’s instructions, either in the absence or presence of CAE. For FCS experiments, SH-SY5Y cells were plated at a density of 4.5 × 10^5^ cells/plate and transfected with eGFP-tagged WT α-Syn/mutant variants and mApple-tagged WT α-Syn/mutant variants for 24/48 h using TurboFect (Thermo Fisher, R0531), as per the vendor’s instructions, in the absence or presence of CAE. Before confocal imaging and FCS measurements, the medium was replaced with phenol red-free DMEM (Gibco, 31053–028). During data acquisition, cells were maintained at 37 °C in a saturated humidity atmosphere containing 95% air and 5% CO_2_ in a culture dish microincubator (Warner, DH-40iL).

### Cell Viability Assay

SH-SY5Y cells either expressing or not expressing α-Syn were seeded in 96-well plates (α-plus, 116196–1SS) at a density of 3.5 × 10^4^ cells/well and treated with various concentrations of CAE, such as WP (500, 700, 900, 1100, 1300, 1500, 1700, and 1900 μg/mL) and OP (5, 10, 50, 75, 100, 150, and 200 μg/mL), at 37 °C for 24 h. Cell viability was assessed using the MTT (3-(4,5-dimethylthiazole-2-yl)-2,5-diphenyltetrazolium bromide) assay (Alfa Aesar, L11939). After 24 h of transfection, the medium was removed, and the cells were washed with phosphate buffer (137 mM NaCl, 2.7 mM KCl, 10 mM Na_2_HPO_4_, and 1.8 mM KH_2_PO_4_). Then, DMEM containing MTT (0.5 mg/mL) was added, and the cells were incubated at 37 °C in a humidified atmosphere containing 95% air and 5% CO_2_ for 4 h. After incubation, the solution was removed, and the cells were washed with phosphate buffer (137 mM NaCl, 2.7 mM KCl, 10 mM Na_2_HPO_4_, and 1.8 mM KH_2_PO_4_). The formazan crystals were then solubilized in 300 μL of DMSO (Cyrusbioscience, 101–67–68–5), and the absorbance was measured at 570 nm using a microplate reader (SpectraMax i3, Molecular Devices). The difference between each group was analyzed using a paired *t*-test.

### Detection of Intracellular ROS (H_2_O_2_) Generation

SH-SY5Y cells either expressing or not expressing α-Syn were plated at a density of 3.5 × 10^4^ cells/well in 96-well plates (α-plus, 116196–3SB) at 37 °C for 24 h. To measure intracellular hydrogen peroxide (H_2_O_2_), the OxiVision Green hydrogen peroxide sensor (AAT Bioquest, 11,503) was preincubated with the cells for 1 h before CAE treatment (OP: 55 μg/mL, WP: 1000 μg/mL) for an additional 80 min at 37 °C. The fluorescence intensity inside the cells was measured using the microplate reader (Molecular devices, SpectraMax iD3) with an excitation wavelength of 490 nm and an emission wavelength of 525 nm.

### Mitochondrial Membrane Potential Assay

SH-SY5Y cells either expressing or not expressing α-Syn were plated at a density of 3.5 × 10^4^ cells/well in 96-well plates (α-plus, 116196–3SB at 37 °C for 24 h. To measure the mitochondrial membrane potential, cells were preincubated with 100 μL of JC-1 (Abcam, ab113850) for 10 min. Subsequently, the cells were washed twice with 1X dilution buffer before CAE treatment (OP: 55 μg/mL, WP: 1000 μg/mL) for a further 150 min at 37 °C. The fluorescence intensity inside the cells was measured using the microplate reader (Molecular devices, SpectraMax iD3) with an excitation wavelength of 475 nm and an emission wavelength of 550 nm for monomeric JC-1 and with an excitation wavelength of 535 nm and an emission wavelength of 590 nm for oligomeric JC-1.

### ATP Assay

SH-SY5Y cells, with or without expressing α-Syn, were plated at a density of 3.5 × 10^4^ cells/well in 96-well plates (α-plus, 116196–3SB) at 37 °C for 24 h. To measure the ATP level, cells were treated with CAE (OP: 55 μg/mL, WP: 1000 μg/mL) for 150 min at 37 °C. Then, 50 μL of detergent (supplied by vender) was added and shaken for 5 min, followed by the addition of 50 μL of substrate solution (supplied by vender) and further shaking for 5 min. The cells were then left to incubate in the dark for 10 min. Luminescence intensity was measured by using a microplate reader (Molecular devices, SpectraMax iD3). Statistical analysis was performed using a paired *t*-test to compare the differences between each pair of groups.

### Caspase-1/Caspase-3 Activity Detection

Cells (1.0 × 10^6^), either expressing or not expressing α-Syn, were treated with CAE (OP: 55 μg/mL, WP: 1000 μg/mL) for 24 h at 37 °C before being lysed with 50 μL of lysis buffer (supplied by vender). Protein concentrations were determined using the Bradford method (Scientific Biotech Corp, BR01–500). Caspase-1and caspase-3 activities were detected using assay kits ab273268 (Abcam) and K106–25 (BioVision), respectively. Next, 150 μg of total protein was treated with 50 μL of 2X reaction buffer (supplied by vender) and 5 μL of the 4 mM YVAD-pNA/DEVD-pNA substrate (200 μM final concentration) and incubated for 2 h at 37 °C before recording the absorbance at 400 nm using a microplate reader (Molecular devices, SpectraMax iD3). The percentage changes in caspase-1/caspase-3 activity were calculated based on the OD_400_ ratio. Paired *t* tests were used to analyze the differences between each pair of groups.

### Autophagy Activity Detection

SH-SY5Y cells, with or without α-Syn expression, were seeded at a density of 3.5 × 10^4^ cells/well in 96-well plates (α-plus, 116196–3SB). To measure autophagy activity, cells were exposed to CAE (OP: 55 μg/mL; WP: 1000 μg/mL) for 4 h at 37 °C. After the treatment, cells were incubated with the detection reagent (supplied by vender) for 30 min. Following the removal of the reagent, 100 μL of 1X assay buffer was added, and fluorescence intensity was measured using a microplate reader (Molecular devices, SpectraMax iD3) with excitation and emission wavelengths of 480 and 530 nm, respectively. A paired *t*-test was used to analyze the difference between each pair of groups.

### Immunofluorescence Analysis

SH-SY5Y cells, with or without α-Syn expression, were seeded on 22 mm coverslips (Marienfeld, AP-0111620) in a 12-well plate (α-plus, 16112) at a density of 6.0 × 10^4^ cells/well. After the transfection, the cells were treated with CAE (OP: 55 μg/mL, WP: 1000 μg/mL) for 4 h at 37 °C. Following the treatment, the cells were fixed with 4% paraformaldehyde (ALFA, A11313) and permeabilized with 0.1% Triton X-100 (Sigma-Aldrich, ×100) for 10 min. After blocking with 5% bovine serum albumin (BioShop, ALB001) for 30 min, the cells were incubated with 10 μg/mL antibody of LC3B (Abcam, ab192890) at room temperature (RT) for 1 h, followed by incubation with 4 μg/mL Goat anti-Rabbit IgG H&L (Alexa Fluor 488, Abcam, ab11150081) at RT for 1 h. The nuclei were stained with 0.5 μg/mL 4′,6-siamidino-2-phenylindole (DAPI, Abcam, ab150081). Confocal fluorescence microscopy was used to examine the cells.

### Fluorescence Correlation Spectroscopy Experiments

FCS was conducted using a custom confocal system based on a Nikon Ti Eclipse, utilizing 470 nm (Picoquant, LDH–P–C-470M) and 560 nm (Picoquant, LDH-D-TA-560B) lasers to excite the sample. The lasers were directed through a 405/488/561/635 nm dichroic mirror (Semrock, Di01-R405/488/561/635) and focused onto the sample using a Nikon Apochromat 100× objective (NA 1.40, oil). Fluorescence emission was collected through a 405/488/561/635 nm notch filter (Semrock, NF03–405/488/561/635 × 10^–25^) and recorded by using avalanche photodiodes (Picoquant, MPD-5C5T). FCS curves were generated by acquiring 60 s of fluorescence intensity using Symphotime (Picoquant, SP1 + 2). The curves were fitted using either a one-component three-dimensional (3D) free diffusion model or an anomalous model described in eqs 1 and 2, with specific parameters defined for each experimental condition.

Equation 1 describes the correlation function for a 3D diffusion model
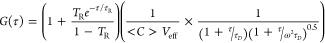


Equation 2 describes the correlation function for an anomalous model
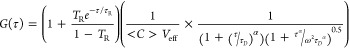


Here, *V*_eff_ denotes the effective excitation volume, characterized by an axial (*z*_0_) to lateral (*r*_0_) dimension ratio *w* ( = *z*_0_*/r*_0_), and <*C*> is the average concentration of the molecules under observation. *T*_R_ and τ_*R*_ refer to the triplet state population and its triplet state relaxation time, respectively, while τ is the correlation time and τ_*D*_ represents the diffusion time. The structure parameter (*w*) and *V*_eff_ are determined through calibrating with a standard dye, R6G (*D* = 414 μm^2^/s).^107^ The anomalous factor α accounts for the effects of the intracellular environment heterogeneity on diffusion.^69,108,109^ We used a 470 nm laser at 15 μW excitation power to excite eGFP and eGFP-tagged α-Syn variants and a 561 nm laser at 40 μW excitation power to excite mApple and mApple-tagged α-Syn variants. To minimize photobleaching and triplet-state blinking, the laser power was adjusted to 25–30 μW during the FCCS experiments.

### Mitochondrial and Lysosome Tracking

For the visualization of mitochondria or lysosomes, SH-SY5Y cells expressing eGPF-α-Syn were incubated with 5 nM MitoTracker Red (Invitrogen) or 5 nM LysoTracker Red (Invitrogen) at 37 °C for 15 min before image acquisition. Confocal image was performed using a homemade confocal system based on a Nikon Ti eclipse. The 470 nm (Picoquant, LDH–P–C-470M) and 635 nm (COHERENT, 9010346) lasers were used to excite eGFP-α-Syn and MitoTracker or LysoTracker, respectively. The overlap between eGFP-α-Syn and mitochondria or lysosomes was quantified by measuring the percentage surface area covered by eGPF-α-Syn, MitoTracker Red, and LysoTracker Red using ImageJ software.

### Statistical Analysis

Data are presented as mean ± SD. Statistical analysis was performed using the unpaired *t*-test with repeated measures. Significant differences were denoted as *, **, and *** for *p* < 0.05, *p* < 0.01, and *p* < 0.001, respectively.

## Supporting Information Available

Cell viability of SH-SY5Y cells with or without expression of WT α-Syn after treatment of cigarette aerosol; cell viability of SH-SY5Y cells with or without expression of WT α-Syn, eGFP, and eGFP α-Syn after treatment of OP CAE and WP CAE; FCS curves and normalized FCCS curves SH-SY5Y cells coexpressing eGFP α-Syn variants and mAppple-α variants with or without treatment of OP CAE for 24 h; FCS curves and normalized FCCS curves SH-SY5Y cells coexpressing eGFP α-Syn variants and mAppple-α variants with or without treatment of WP CAE for 24 h; representative immunofluorescence images of SH-SY5Y cells expressing untagged WT α-Syn with or without treatment of CAE for 24 h; confocal images of SH-SY5Y cells expressing eGFP α-Syn and mAppple-α with or without treatment of OP CAE for 48 h, analysis of mean diameter, synchronization ratio, and diffusion coefficient; confocal images of SH-SY5Y cells expressing eGFP α-Syn and mAppple-α with or without treatment of WP CAE for 24 and 48 h, analysis of mean diameter, synchronization ratio, and diffusion coefficient; and impact of OP CAE on autophagy activities in SH-SY5Y cells with and without the expression of mApple-tagged WT α-Syn or mApple (PDF)

## Abbreviations

CAE: cigarette aerosol extract
Type I: homogeneous distribution region of α-Syn
Type II: puncta region of α-Syn
FCCS: fluorescence cross-correlation spectroscopy
WT: wild type
