# Supplementary material for: Influence of Cigarette Aerosol in Alpha-Synuclein Oligomerization and Cell Viability in SH-SY5Y: Implications for Parkinson’s Disease
Source: ACS Chem Neurosci. 2024 Mar 14;15(7):1484–500. doi: 10.1021/acschemneuro.3c00771 (PMC10995954; doi:10.1021/acschemneuro.3c00771)
Supplement: Supplementary file 1 — cn3c00771_si_001.pdf [file cn3c00771_si_001.pdf]

Supporting information

The Influence of Cigarette Aerosol in Alpha-Synuclein Oligomerization and Cell Viability in SH-SY5Y: Implications for Parkinson's Disease

Yu-Xin Shen[1 2.3+], Pe-Shuen Lee[1 2.3+], Ming-Chu Teng[1 2.3], Jhih-Hong Huang[2.3], Chia C. Wang[2.3*], Hsiu-Fang Fan[1 2.3*]

[1] Institute of Medical Science and Technology, National Sun Yat-sen University, Kaohsiung, 804, Taiwan

[2] Department of Chemistry, National Sun Yat-sen University, Kaohsiung, 804, Taiwan

[3] Aerosol Science Research Center, National Sun Yat-sen University, Kaohsiung, 804, Taiwan

**** Correspondence can be sent to***

HFF (bendyfan@imst.nsysu.edu.tw) and CCW(chiawang@mail.nsysu.edu.tw)

To be submitted to ACS Chemical Neuroscience

+ These authors have equal contributions to this work

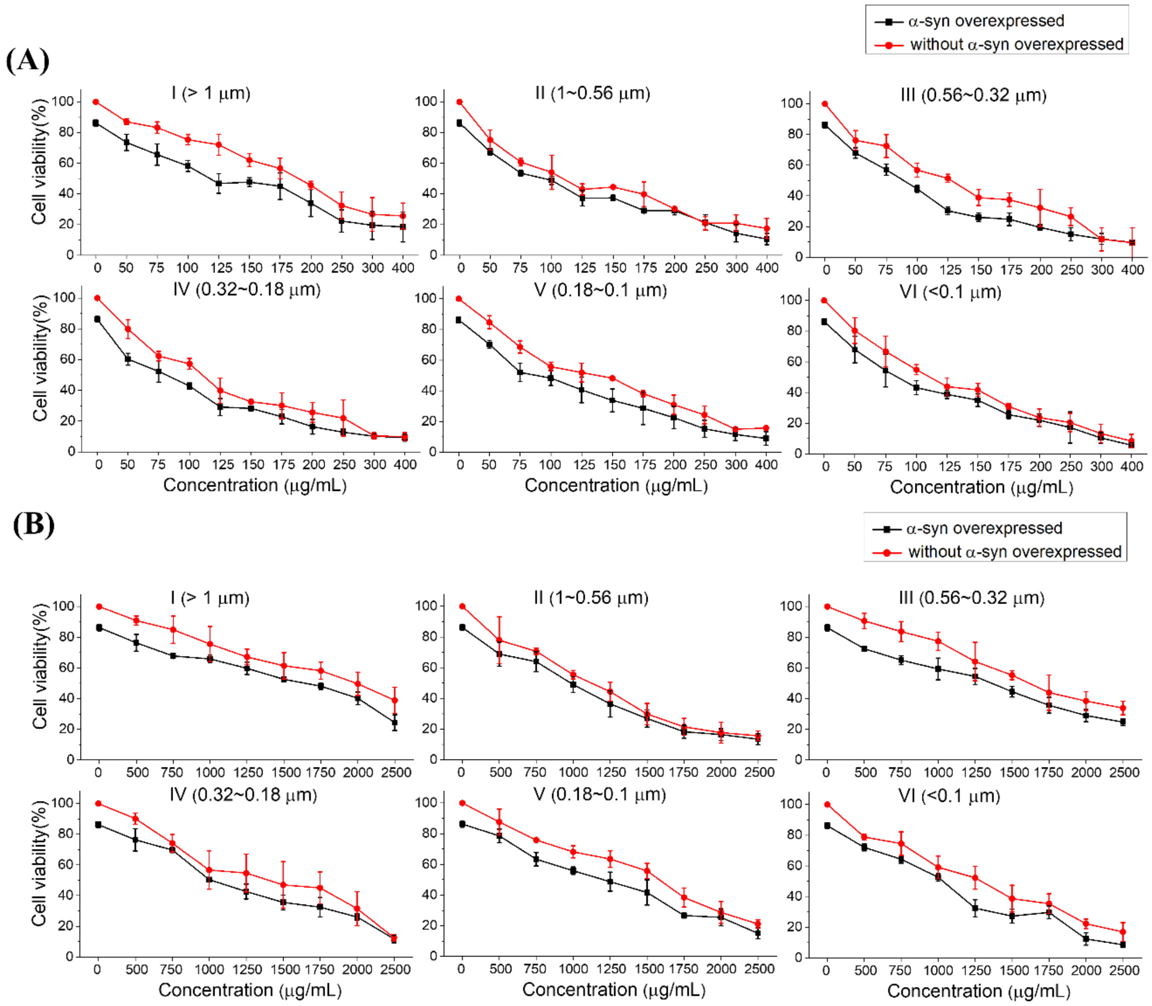

Figure S1 Cell viability of SH-SY5Y cells with or without expressing of WT α-Syn after treatment of cigarette aerosol (A) OP (B) WP extraction for 24 h at 37°C verified by MTT assay. N (repeat of experiment) is 3 for each condition. I~VI indicate the size of cigarette aerosol obtained with MOUDI listed in Supplementary Table 1.

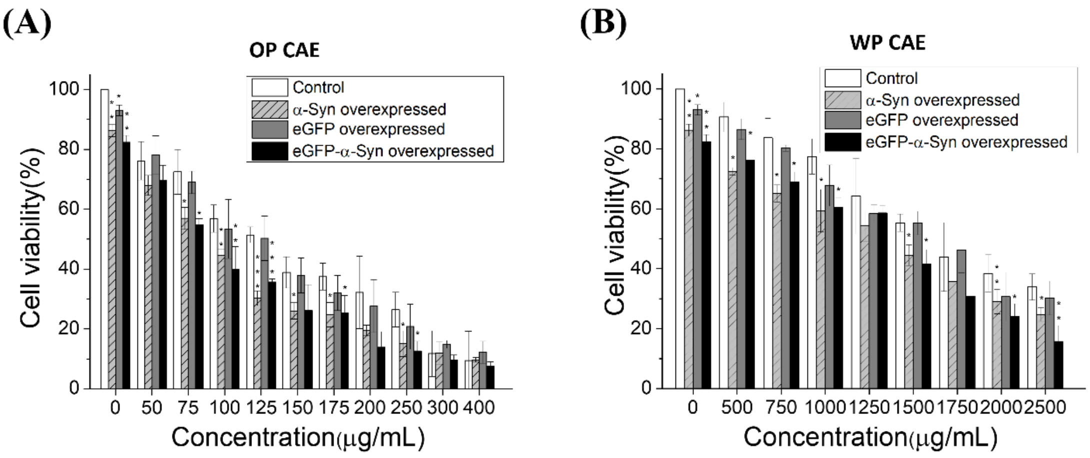

Figure S2 Cell viability of SH-SY5Y cells with or without expressing of WT α-Syn, eGFP and eGFP α-Syn after treatment of (A) OP CAE, $IC_{50,Control}$ = 124.60 μg/mL; $IC_{50,\alpha\text{-}Syn}$ = 92.92 μg/mL; $IC_{50,eGFP}$ = 120.64 μg/mL; $IC_{50,\ eGFP\ \alpha\text{-}Syn}$ = 85.42 μg/mL. (B) WP CAE, $IC_{50,Control}$ = 1653.95 μg/mL; $IC_{50,\alpha\text{-}Syn}$ = 1476.23 μg/mL; $IC_{50,eGFP}$ = 1612.06μg/mL; $IC_{50,\ eGFP\ \alpha\text{-}Syn}$ = 1458.37 μg/mL. for 24 h at 37°C verified by MTT assay. N (repeat of experiment) is 3 for each condition.

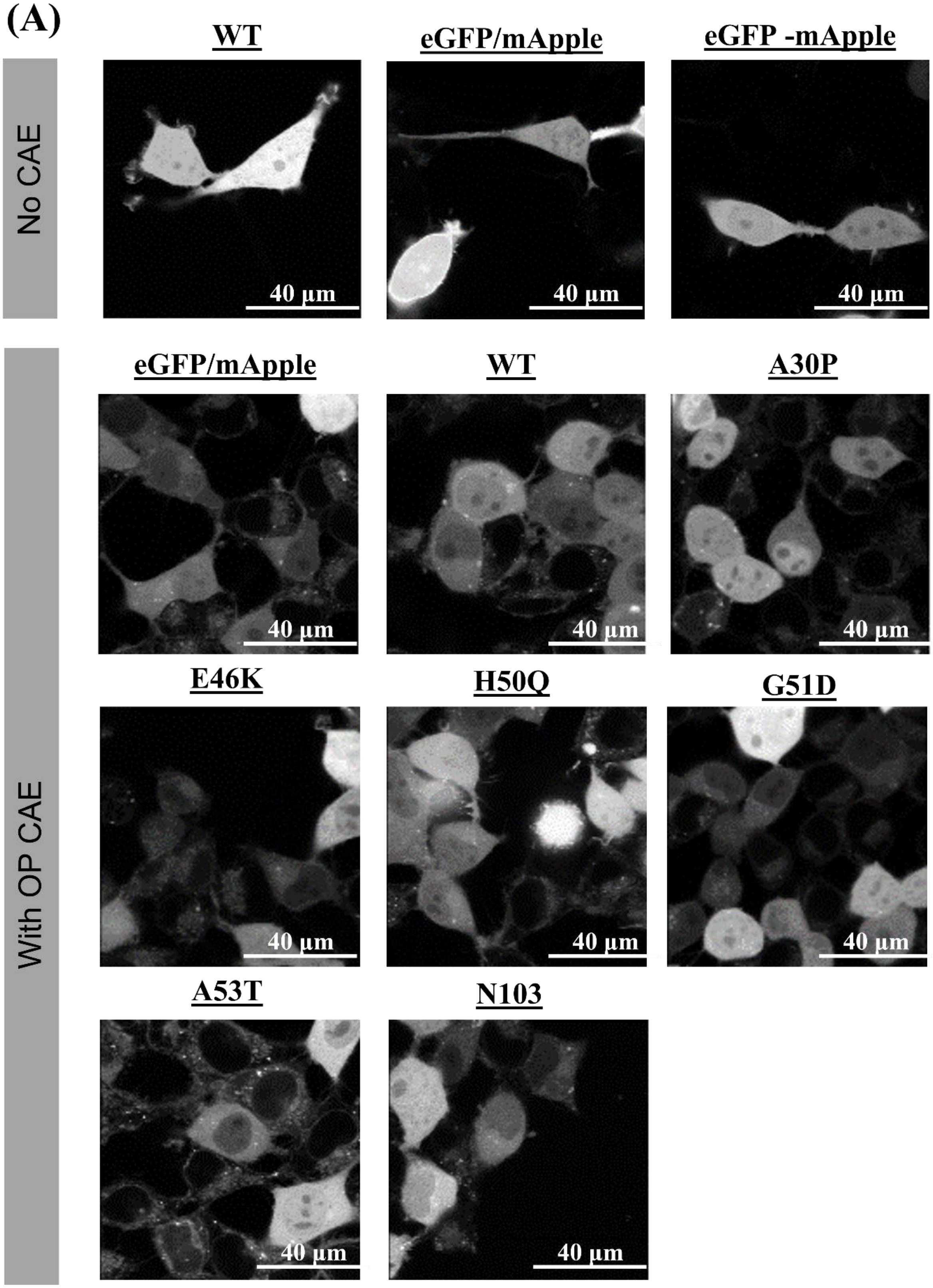
(A)
No CAE
WT
eGFP/mApple
eGFP -mApple
With OP CAE
eGFP/mApple
WT
A30P
E46K
H50Q
G51D
A53T
N103
40 µm

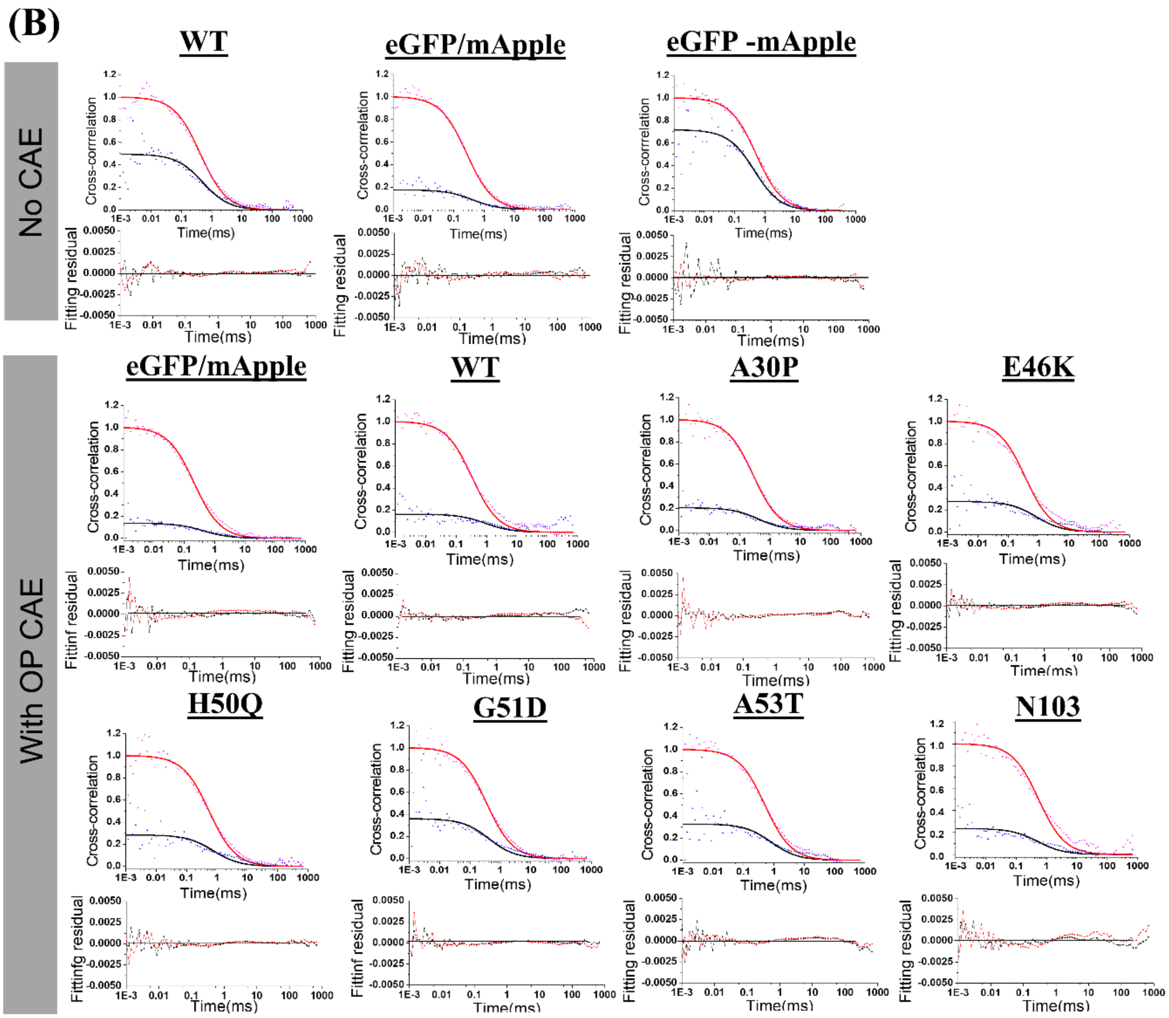
(B)
WT
eGFP/mApple
eGFP -mApple
No CAE
With OP CAE
eGFP/mApple
WT
A30P
E46K
H50Q
G51D
A53T
N103
Cross-correlation
Fitting residual
Time(ms)

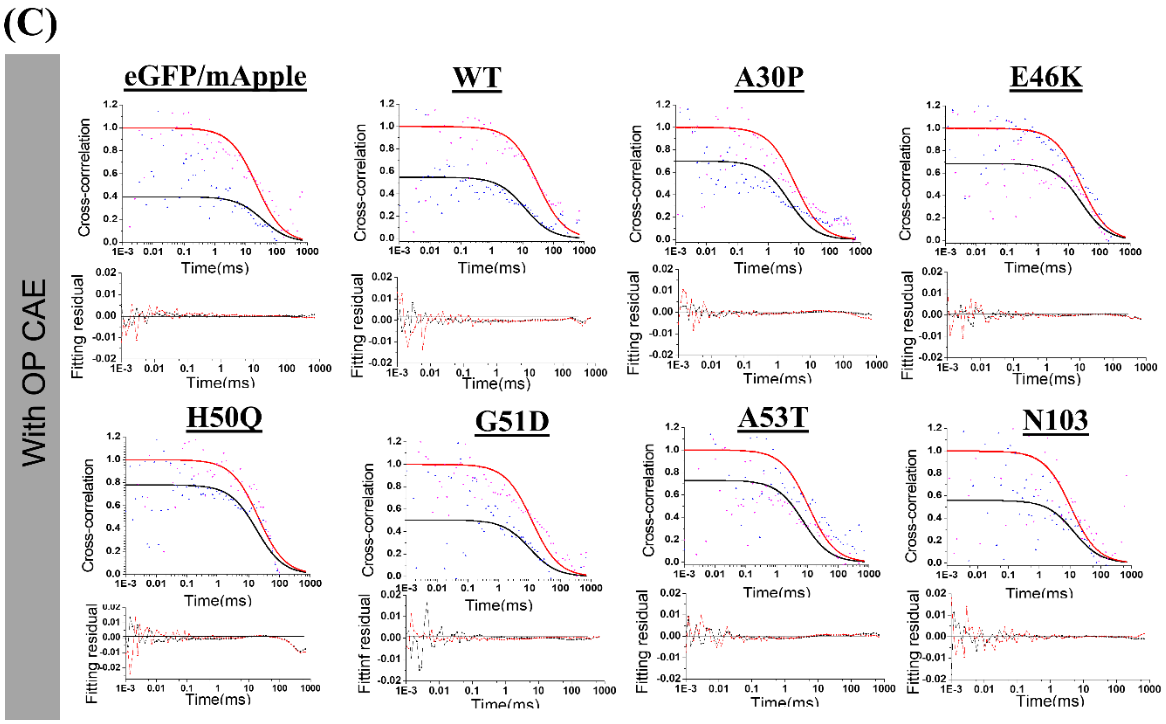

Figure S3. FCS curve and normalized FCCS curve of SH-SY5Y cells co-expressing eGFP-α Syn variants and mApple-α Syn variants with or without treatment of OP CAE for 24 h at 37°C. Data acquired in (A) homogeneous distribution region and **(B)** puncta regions. Each FCS curve was fitted with one-component 3D free diffusion model to obtain the corresponding diffusion coefficient (Listed in Table I).

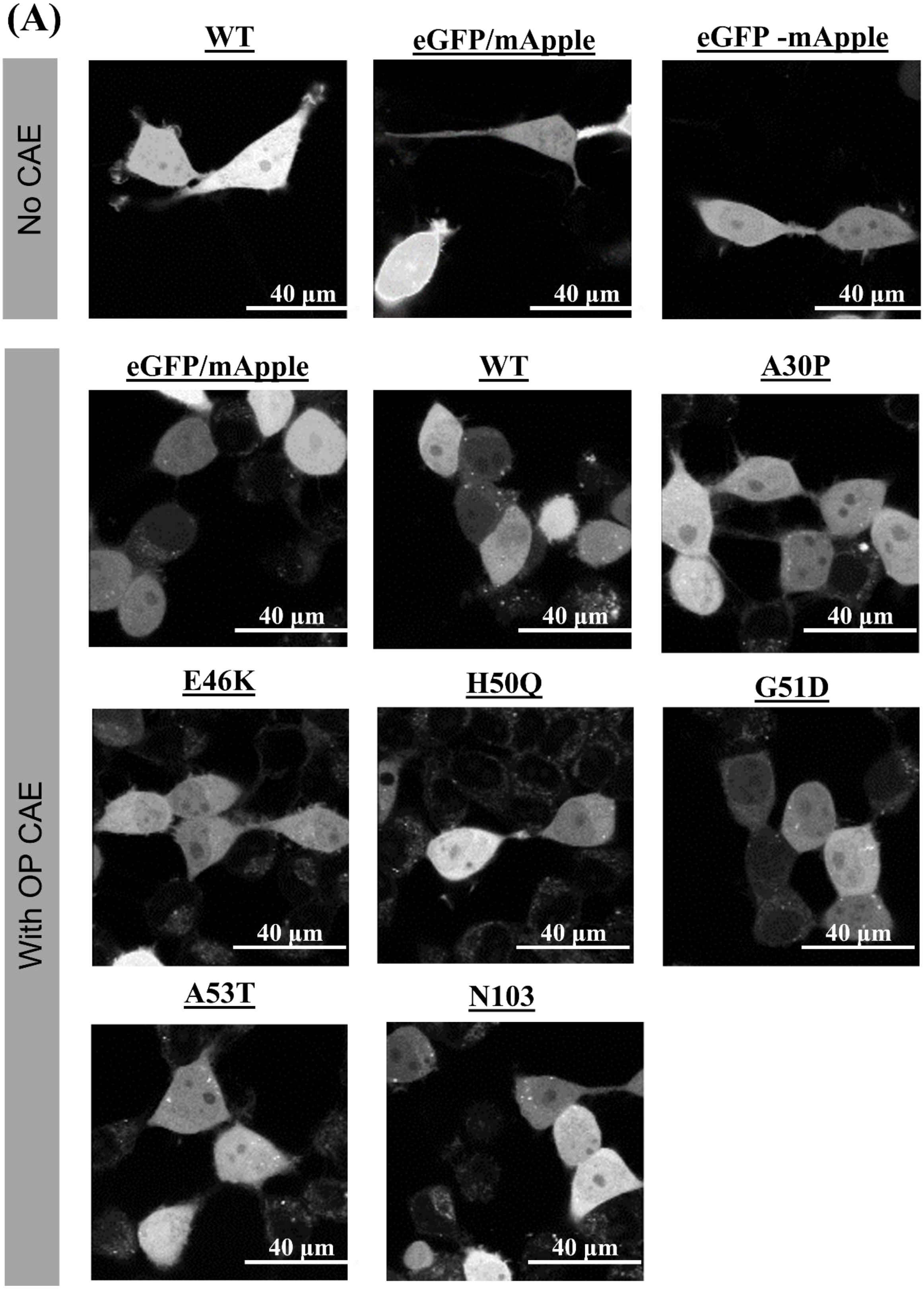
(A)
WT
eGFP/mApple
eGFP -mApple
No CAE
40 µm
40 µm
40 µm
With OP CAE
eGFP/mApple
WT
A30P
40 µm
40 µm
40 µm
E46K
H50Q
G51D
40 µm
40 µm
40 µm
A53T
N103
40 µm
40 µm

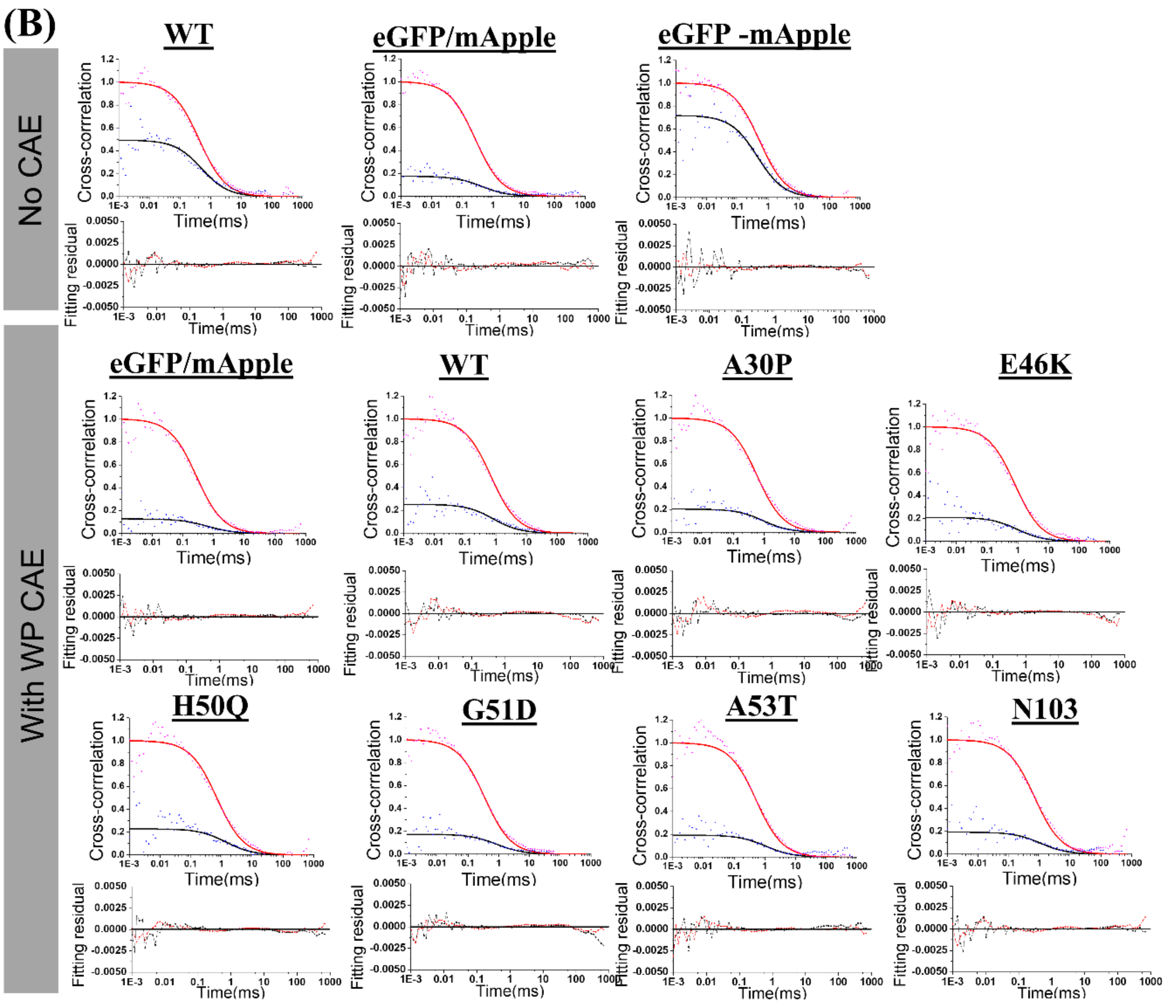
(B)
No CAE
With WP CAE
WT
eGFP/mApple
eGFP -mApple
eGFP/mApple
WT
A30P
E46K
H50Q
G51D
A53T
N103
Cross-correlation
Fitting residual
Time(ms)

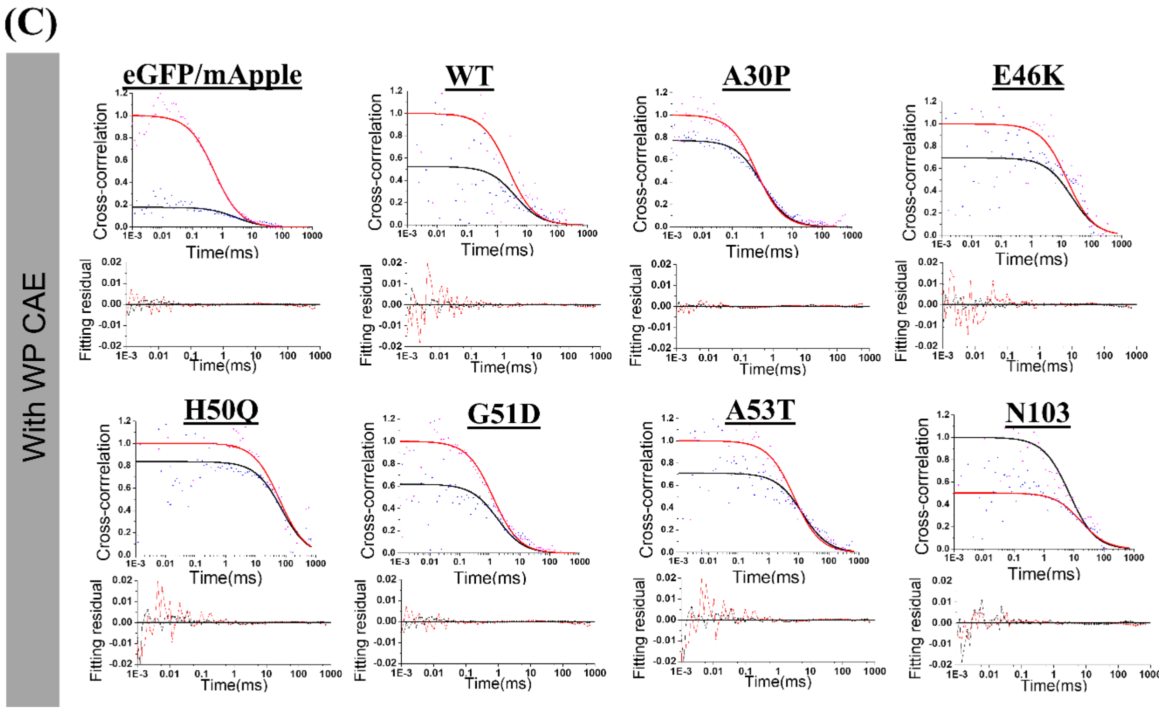

Figure S4 FCS curve and normalized FCCS curve of SH-SY5Y cells co-expressing eGFP-α Syn variants and mApple-α Syn variants with or without treatment of WP CAE for 24 h at 37°C. Data acquired in (A) homogeneous distribution region and **(B)** puncta regions. Each FCS curve was fitted with one-component 3D free diffusion model to obtain the corresponding diffusion coefficient (Listed in Table II).

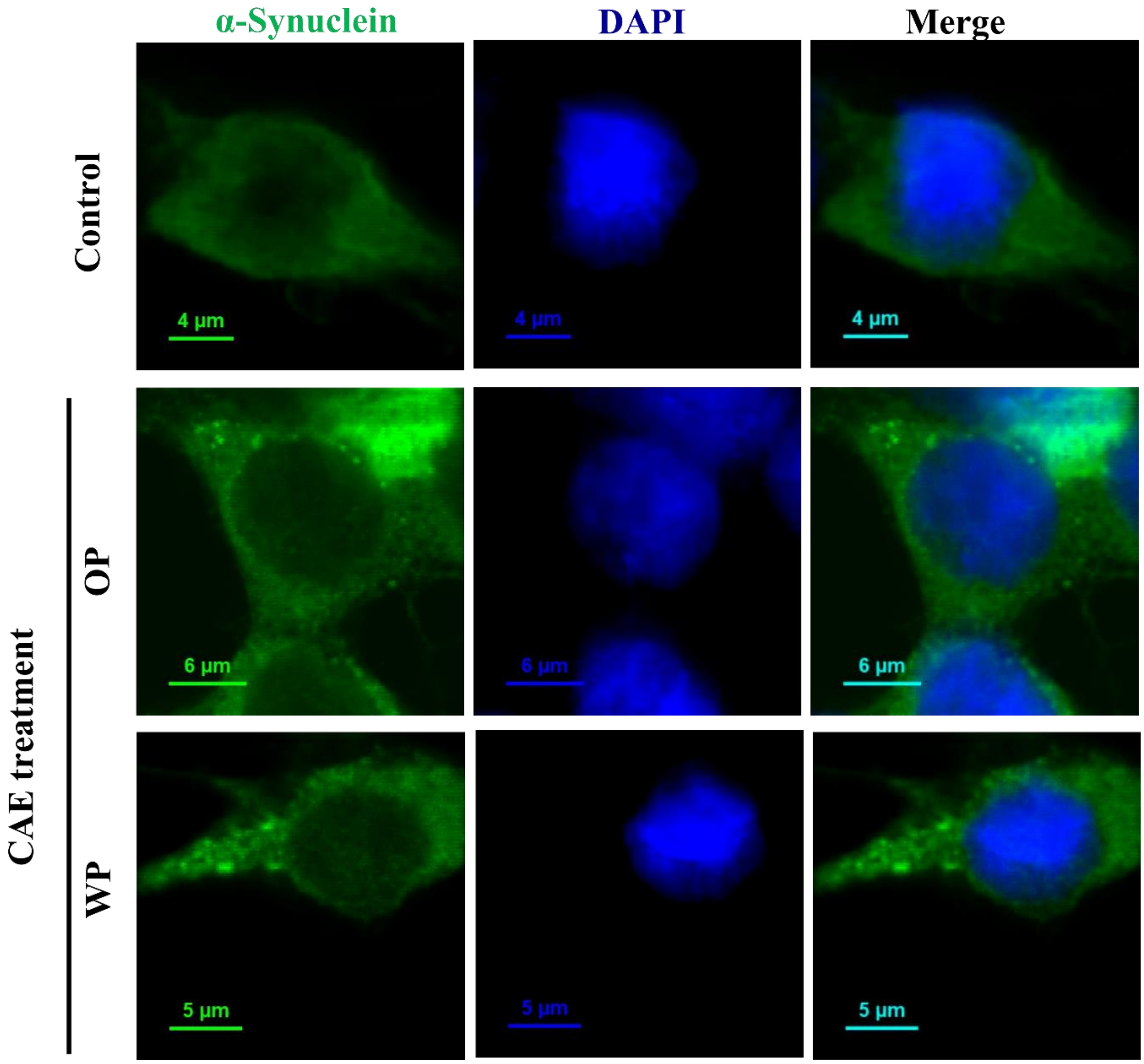

Figure S5. Representative immunofluorescence images of SH-SY5Y cells expressing untagged WT α-Syn with or without treatment of CAE (OP: 20 μg/mL, WP: 200 μg/mL) for 24 h.

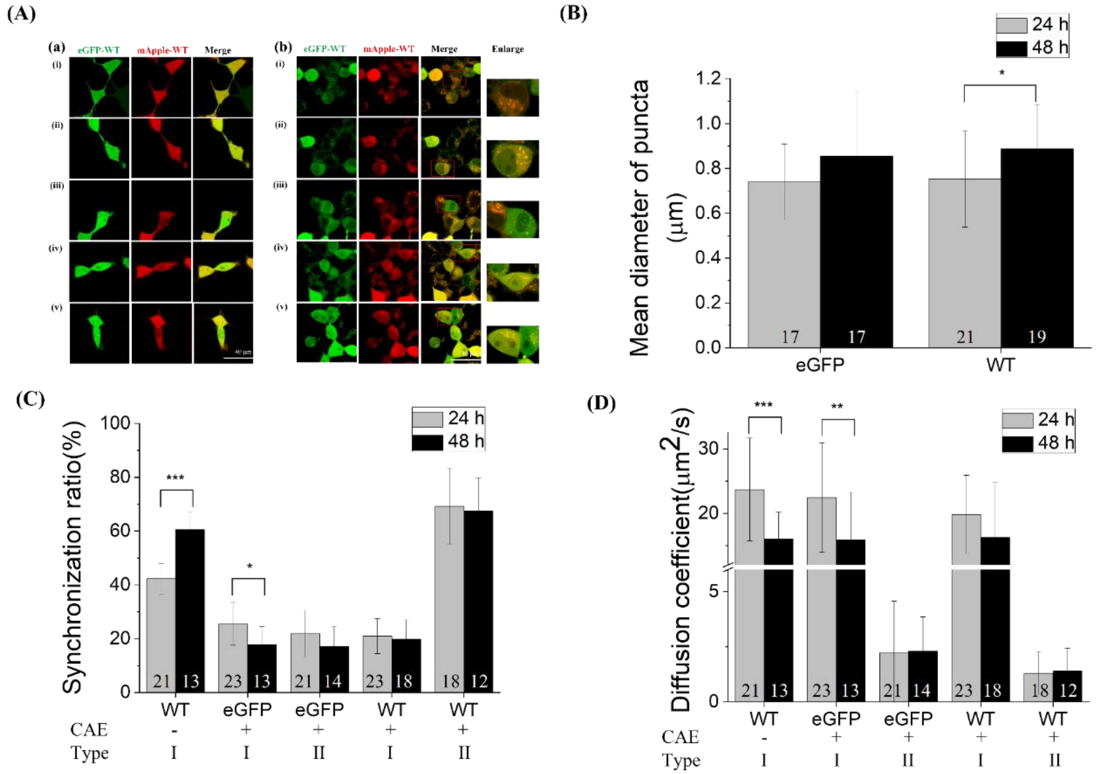
(A)
(a)
eGFP-WT
mApple-WT
Merge
(i)
(ii)
(iii)
(iv)
(v)
(b)
eGFP-WT
mApple-WT
Merge
Enlarge
(B)
24 h
48 h
Mean diameter of puncta (μm)
1.2
1.0
0.8
0.6
0.4
0.2
0.0
*
17
17
21
19
eGFP
WT
(C)
Synchronization ratio(%)
100
80
60
40
20
0
24 h
48 h
***
*
21
13
23
13
21
14
23
18
18
12
WT
eGFP
eGFP
WT
WT
CAE
-
+
+
+
+
Type
I
I
II
I
II
(D)
Diffusion coefficient(μm2/s)
30
20
5
0
***
**
24 h
48 h
21
13
23
13
21
14
23
18
18
12
WT
eGFP
eGFP
WT
WT
CAE
-
+
+
+
+
Type
I
I
II
I
II

Figure S6. (A). Confocal images of SH-SY5Y cells expressing eGFP-α Syn and mApple-α Syn with or without treatment of OP-CAE for 48 h. **(B)**. Analysis of mean diameter of puncta from SH-SY5Y cells expressing eGFP-α Syn and mApple-α Syn with treatment of OP-CAE for 24 and 48 h. **(C)** Synchronization (cross-correlation) ratio of eGFP-α Syn and mApple-α Syn in SH-SY5Y cells with treatment of OP-CAE for 24 and 48 h. (**D)**. Diffusion coefficient of eGFP-α Syn and mApple-α Syn SH-SY5Y cells with treatment of OP-CAE for 24 and 48 h. Each FCS curve was fitted with one-component 3D free diffusion model to obtain the corresponding diffusion coefficient (Listed in Table 3). *, **, and *** represent significant differences (* = $p < 0.05$), (** = $p < 0.01$) and (*** = $p < 0.001$). The numbers indicate the number of investigated cells.

(A)

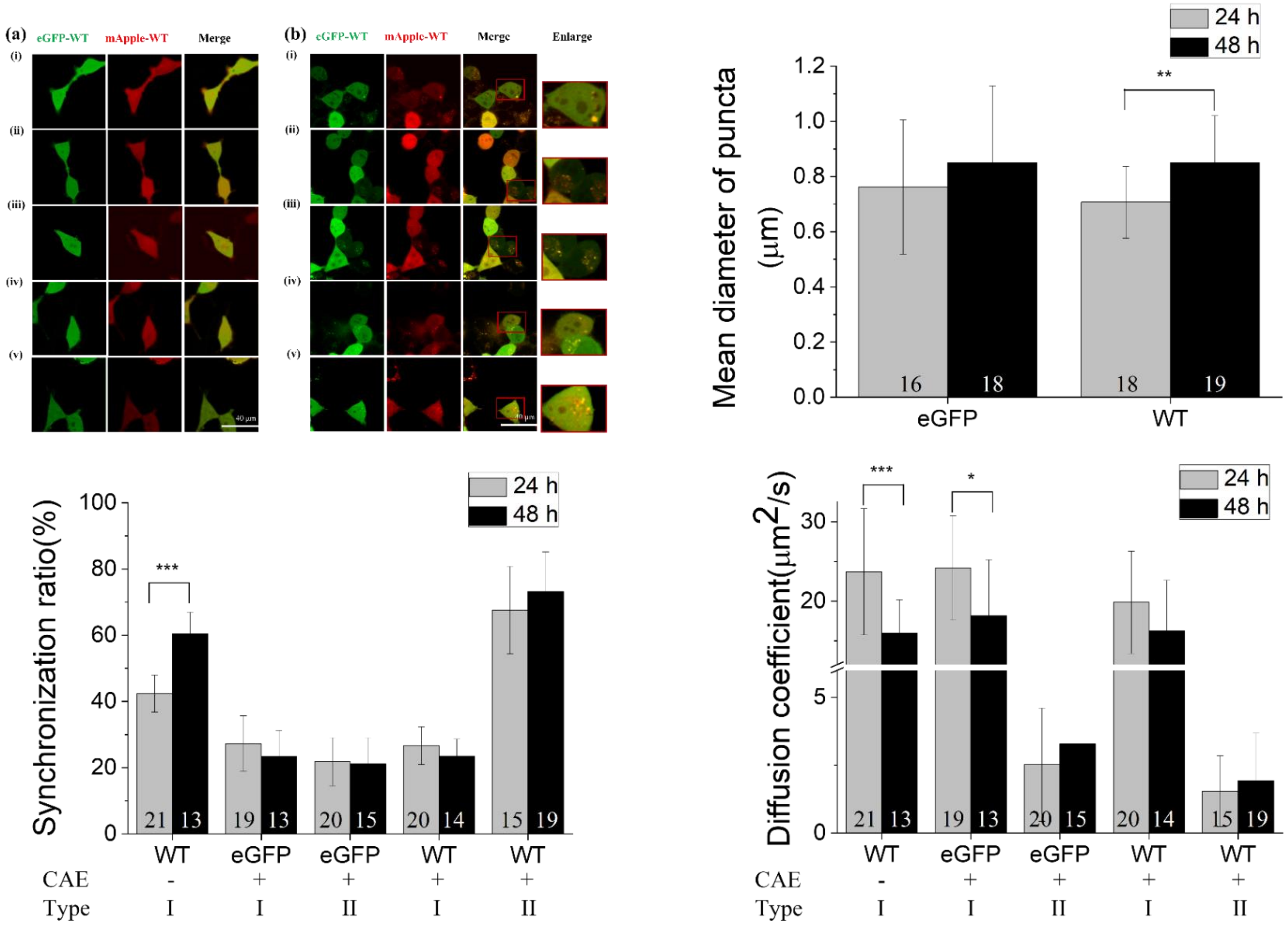

(a)
eGFP-WT
mApple-WT
Merge
(i)
(ii)
(iii)
(iv)
(v)
40 μm
(b)
eGFP-WT
mApple-WT
Merge
Enlarge
Mean diameter of puncta (μm)
24 h
48 h
**
16
18
18
19
eGFP
WT
Synchronization ratio(%)
***
21 13
19 13
20 15
20 14
15 19
WT
eGFP
eGFP
WT
WT
CAE
Type
Diffusion coefficient(μm2/s)
***
*

Figure S7. (A). Confocal images of SH-SY5Y cells expressing eGFP-α Syn and mApple-α Syn with or without treatment of WP-CAE for 24 and 48 h. **(B)**. Analysis of mean diameter of puncta from SH-SY5Y cells expressing eGFP-α Syn and mApple-α Syn with treatment of WP-CAE for 48 h. **(C)** Synchronization (cross-correlation) ratio of eGFP-α Syn and mApple-α Syn in SH-SY5Y cells with treatment of WP-CAE for 24 and 48 h. (**D)**. Diffusion coefficient of eGFP-α Syn and mApple-α Syn SH-SY5Y cells with treatment of WP-CAE for 24 and 48 h. Each FCS curve was fitted with one-component 3D free diffusion model to obtain the corresponding diffusion coefficient (Listed in Table 3). *, **, and *** represent significant differences (* = $p < 0.05$), (** = $p < 0.01$) and (*** = $p < 0.001$). The numbers indicate the number of investigated cells.

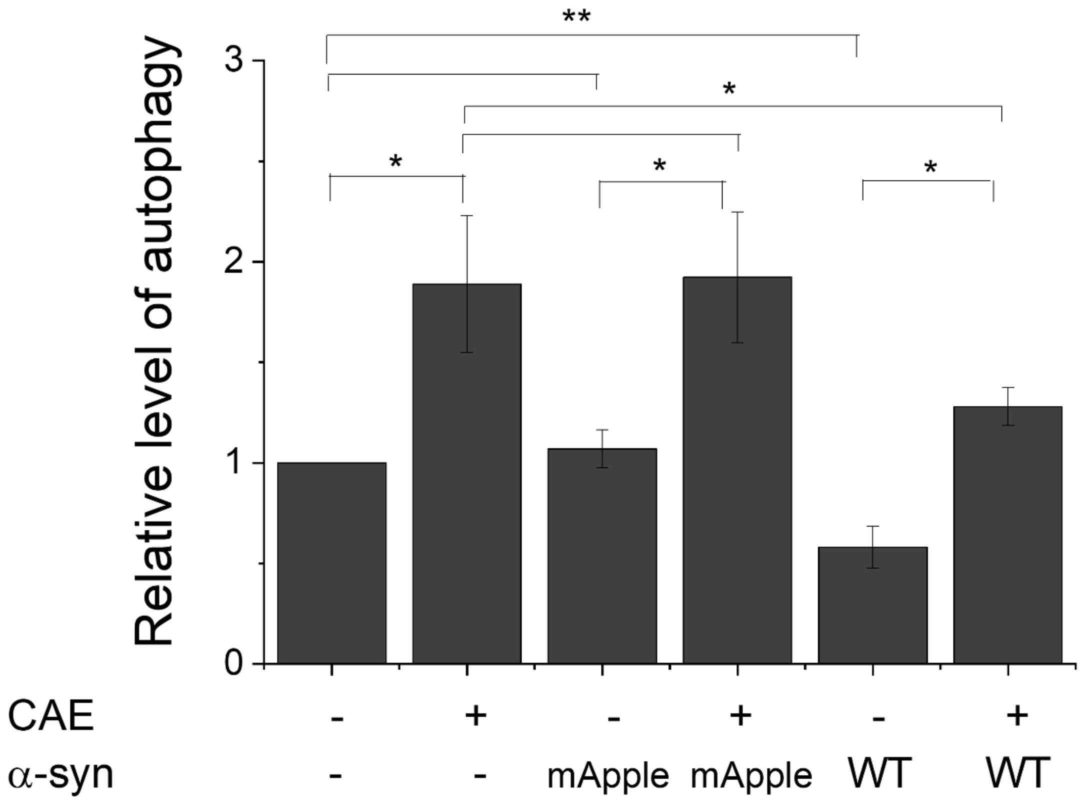

Figure S8. The impact of OP-CAE on autophagy activities in SH-SY5Y cells, both with and without the expression of mApple-tagged WT α-Syn or mApple. *, **, and *** represent significant differences (* = $p < 0.05$), (** = $p < 0.01$) and (*** = $p < 0.001$). The signal representing autophagy activity is overlapped with the emission spectrum of eGFP; therefore, mApple instead of eGFP was used here to verify the influence of fluorescence protein overexpression on autophagy activity. mApple represents cells overexpressing mApple. WT represents cells overexpressing mApple-tagged WT α-Syn. "–" represents control cells without overexpressing.

Supplementary Table 1. The classification of the size of cigarette aerosol obtained with MOUDI

	Label					
	I	II	III	IV	V	VI
Diameter of PM (μm)	>1	1~0.56	0.56~0.32	0.32~0.18	0.18~0.1	<0.1
